# Global Metabolomics of the Placenta Reveals Distinct Metabolic Profiles between Maternal and Fetal Placental Tissues Following Delivery in Non-Labored Women

**DOI:** 10.3390/metabo8010010

**Published:** 2018-01-23

**Authors:** Jacquelyn M. Walejko, Anushka Chelliah, Maureen Keller-Wood, Anthony Gregg, Arthur S. Edison

**Affiliations:** 1 Department of Biochemistry & Molecular Biology, University of Florida, Gainesville, FL 32610, USA; jwalejko@ufl.edu; 2 Department of Obstetrics, Gynecology, and Reproductive Sciences, University of Texas Health Science Center at Houston, UT Health, Houston, TX 77030, USA; anushka.chelliah@uth.tmc.edu; 3 Department of Pharmacodynamics, University of Florida, Gainesville, FL 32610, USA; kellerwd@cop.ufl.edu; 4 Department of Obstetrics and Gynecology, University of Florida, Gainesville, FL 32610, USA; greggar@ufl.edu; 5 Departments of Genetics and Biochemistry & Molecular Biology, Institute of Bioinformatics, Complex Carbohydrate Research Center, University of Georgia, Athens, GA 30602, USA

**Keywords:** pregnancy, human, placenta, metabolomics, ^1^H-NMR

## Abstract

We evaluated the metabolic alterations in maternal and fetal placental tissues from non-labored women undergoing cesarean section using samples collected from 5 min to 24 h following delivery. Using ^1^H-NMR, we identified 14 metabolites that significantly differed between maternal and fetal placental tissues (FDR-corrected *p*-value < 0.05), with 12 metabolites elevated in the maternal tissue, reflecting the flux of these metabolites from mother to fetus. In the maternal tissue, 4 metabolites were significantly altered at 15 min, 10 metabolites at 30 min, and 16 metabolites at 1 h postdelivery, while 11 metabolites remained stable over 24 h. In contrast, in the fetal placenta tissue, 1 metabolite was significantly altered at 15 min, 2 metabolites at 30 min, and 4 metabolites at 1 h postdelivery, while 22 metabolites remained stable over 24 h. Our study provides information on the metabolic profiles of maternal and fetal placental tissues delivered by cesarean section and reveals that there are different metabolic alterations in the maternal and fetal tissues of the placenta following delivery.

## 1. Introduction

The placenta is an organ that develops during pregnancy and has numerous functions in the mother and fetus, such as preventing rejection of the fetal allograft, transporting nutrients, eliminating waste products, and enabling gas exchange. Sheep models of pregnancy have found that 40–80% of all oxygen and glucose that reach the placenta are utilized to produce fructose, nonessential amino acids, and lactate, which are then delivered to the fetus as nutrients [1,2,3,4,5,6,7]. However, these metabolic pathways have not been well characterized in the human placenta. Several studies utilized stable isotope tracers in women to determine the flux of various amino acids across the placenta in late gestation prior to delivery [8,9,10,11]. In addition, cells from the human placenta revealed a higher propensity for aerobic glycolysis, while metagenomic sequencing from the surface of the placenta revealed a distinct microbiome environment, with several microbes that have metabolic functions in the placenta [12,13]. Metabolic pathways of the human placenta were identified by transcriptomic analysis, but the related downstream metabolites have not yet been studied in depth [14,15].

Metabolomics is the study of small molecules in a biological system and has been utilized to study both placental cells and whole placental tissue in pregnancies complicated by disease [16,17,18,19,20,21]. However, there is little information on how timing and area of collection affect metabolic profiles in placental tissue. The human placenta is made up of two components: a maternal surface that develops from the endometrium and a fetal surface that develops from fetal cells [22]. To date, there is little information on metabolic differences between specimens collected from the maternal or fetal surfaces of the placenta and on whether the stability of metabolites differs between these tissue types as a function of time before extraction. The current data for the optimization of placental specimen collection for metabolomics studies recommends immediate processing of vaginally delivered specimens, within 10 min of delivery [23]. While this short time is ideal, it poses a significant practical challenge for specimen collection in the delivery room, and the lack of timely processing may result in the loss of useful metabolic data. In addition, the optimal time following delivery to collect specimens for metabolomics studies has not been reported for women that undergo non-labored, cesarean delivery. In this study, we evaluated the differences in metabolites measured in maternal and fetal placental specimens, as well as the changes in these metabolites at various time points from immediate up to 24 h postdelivery in women undergoing non-labored cesarean deliveries.

## 2. Results

### 2.1. Metabolic Alterations between the Maternal and Fetal Surfaces of the Placenta

**Figure 1:**
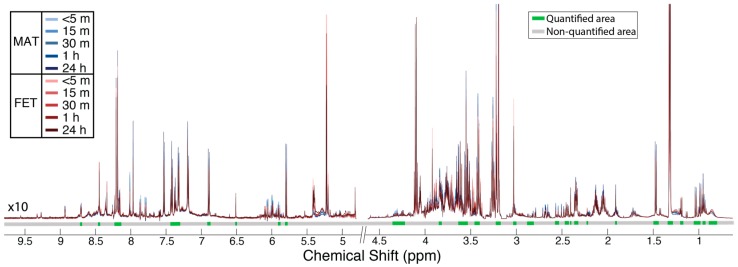
Overlay of mean ^1^H-NMR spectra from five time points following placenta delivery: (1) <5 min (*n* = 24), (2) 15 min (*n* = 49), (3) 30 min (*n* = 51), (4) 1 h (*n* = 51), and (5) 24 h (*n* = 51). Maternal spectra (MAT) are displayed in blue, while fetal (FET) spectra are displayed in red. Metabolite identification was conducted using 2D NMR data (HSQC and HSQC–TOCSY), using COLMARm and Bruker Assure software. Non-overlapped peaks for the identified metabolites (*n* = 38) are shown in green under the spectra, while areas not quantified are displayed in gray. The negative tails of peaks seen around some resonances are from Carr–Purcell–Meiboom–Gill (CPMG) pulse sequence artifacts arising from antiphase coherence.

**Figure 2:**
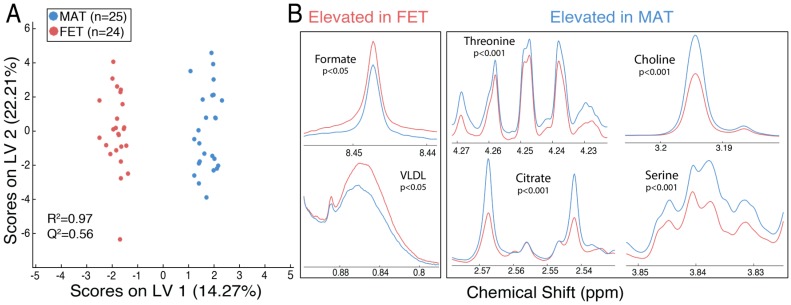
(**A**) Orthogonal signal correction–partial least squares discriminant analysis (OSC–PLSDA) scores plot reveals a separation of MAT (blue, *n* = 25) and FET (red, *n* = 24) spectra from 13 placentas at 15 min postdelivery; (**B**) the mean spectra of FET (red) and MAT (blue) display significant (FDR-corrected *p* < 0.05) elevations in formate and VLDL in fetal placental specimens (left), while threonine, choline, citrate, and serine are significantly elevated in the maternal tissue (right). Table 1 displays the means with standard errors, FDR-corrected p-value, and fold change for all metabolites identified.

Figure 1 shows the normalized proton nuclear magnetic resonance (^1^H-NMR) spectra from maternal (blue) and fetal (red) placental specimens at five time points following delivery, as indicated by shading (light < 5 min to dark at 24 h). The metabolite areas used for quantitation are indicated by green bars under the spectra, while the regions that were not quantified are indicated by grey bars. Orthogonal signal correction–partial least squares discriminant analysis (OSC–PLSDA) revealed a separation between maternal and fetal placental specimens (Figure 2a; Component 1, R^2^Y = 0.97, Q^2^Y = 0.56). A principal component analysis (PCA) of the same data showed some separation but was less useful (Supplementary Material Figures S1–S3). At 15 min postdelivery, many metabolites were significantly elevated (FDR-corrected *p* < 0.05) in maternal placental specimens, including amino acids (serine and threonine), citrate, and choline (Figure 2b, right). Metabolites significantly elevated (FDR-corrected *p* < 0.05) in fetal placental specimens included very-low-density lipoprotein (VLDL) and formate (Figure 2b, left). All identified metabolites and their corresponding means with standard errors, FDR-corrected *p*-values, and fold changes are listed in Table 1.

### 2.2. The Maternal Surface of the Placenta Reveals Metabolites That Are Sensitive to the Timing of Collection Following Delivery

**Figure 3:**
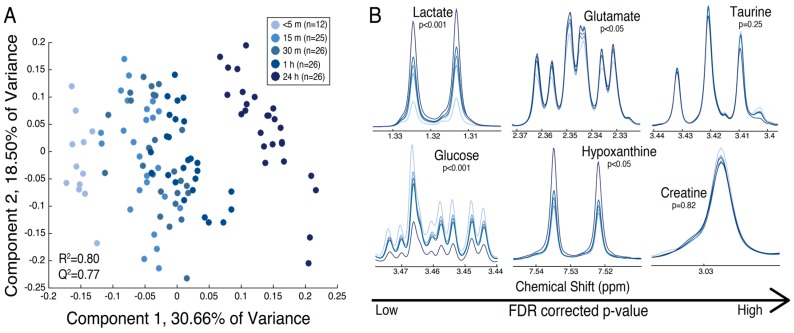
(**A**) The partial least squares discriminant analysis (PLS-DA) scores plot reveals separation of the specimens from the maternal surface of 13 placentas at five time points postdelivery: (1) <5 min (*n* = 12), (2) 15 min (*n* = 25), (3) 30 min (*n* = 26), (4) 1 h (*n* = 26), and (5) 24 h (*n* = 26). The time points are represented as varying shades of blue, from light (<5 min) to dark (24 h); (B) mean spectra of time points displaying metabolites with varying significance of FDR-corrected p-values from left to right (low to high). Lactate significantly increased over time (*p* < 0.001), while glucose diminished (*p* < 0.001). Glutamate and hypoxanthine significantly increased over time (*p* < 0.05), while taurine and creatine did not differ over 24 h. Table 2 displays means with standard errors, FDR-corrected p-value, and fold change between <5 min and 1 h following delivery for all metabolites identified.

Partial least squares discriminant analysis (PLS-DA) revealed separation of the maternal placental specimens based on the time following delivery (Figure 3a). A one-way analysis of variance (ANOVA) revealed that anaerobic glycolysis (elevations in lactate, diminished glucose) occurred within 15 min following delivery, while glutamate and hypoxanthine displayed alterations 1 h and 24 h, respectively, following delivery (FDR-corrected *p* < 0.05). Some metabolites, including taurine and creatine, did not display significant metabolic alterations up to 24 h following delivery in maternal placental specimens (Figure 3b). All metabolites identified in the maternal tissue and their corresponding means with standard errors, FDR-corrected *p*-values, and fold changes over 1 h following delivery are displayed in Table 2. The relative trends for metabolites over 1 h are displayed in Figure S4. Lactate and various amino acids displayed a linear increase over time (Figure S4a), while glycerophosphocholine (GPC), glucose, niacinamide, and formate displayed a linear decrease over time (Figure S4b). The results of Tukey–Kramer post-hoc analysis for each metabolite in the maternal placental tissue are displayed in Table S2.

### 2.3. The Fetal Surface of the Placenta Shows Less Sensitivity to Timing of Collection Following Delivery

**Figure 4:**
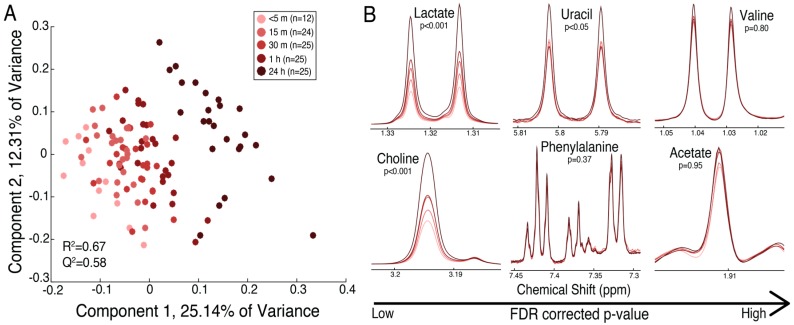
(**A**) The PLS-DA scores plot reveals separation of the specimens from the fetal surface of the placenta from 13 placentas at five time points postdelivery: (1) <5 min (*n* = 12), (2) 15 min (*n* = 24), (3) 30 min (*n* = 25), (4) 1 h (*n* = 25), and (5) 24 h (*n* = 25). The time points are represented as varying shades of red, from light (<5 min) to dark (24 h); (**B**) mean spectra of time points displaying metabolites with varying significance of FDR-corrected *p*-values from left to right (low to high). Lactate, choline, and uracil significantly increased over time (*p* < 0.001), while phenylalanine, valine, and acetate did not show a significant trend over 24 h. Table 3 displays the means with standard errors, FDR-corrected *p*-value, and fold change between <5 min and 1 h following delivery for all metabolites identified.

PLS-DA revealed separation of the fetal placental specimens based on the time of collection following delivery (Figure 4a, left). This separation is not as distinct as that found in maternal placental specimens, as indicated by the poorer model quality (Q^2^ of 0.58 in fetal compared to Q^2^ of 0.88 in maternal). One-way ANOVA revealed significant elevations (FDR-corrected *p* < 0.05) in lactate within 30 min and choline within 1 h following delivery, while uracil was significantly elevated 24 h following delivery. Phenylalanine, acetate, and valine were not significantly altered over time (Figure 4b, right). All metabolites identified in the fetal tissue and their corresponding means with standard errors, FDR-corrected p-value, and fold change over 1 h are displayed in Table 3. The relative trends for metabolites over 1 h are displayed in Figure S5. Similar to the maternal placental metabolite trends, lactate and choline displayed a linear increase over time (Figure S5a), while the storage form of cholines (PC and GPC) and niacinaminde displayed a linear decrease over time (Figure S5b). The results of Tukey–Kramer post-hoc analysis for each metabolite in the fetal placental tissue are displayed in Table S3.

## 3. Discussion

We showed that the maternal and fetal sides of the placenta are metabolically distinct. The concentrations of amino acids, including serine, threonine, aspartate, taurine, and alanine, were greater on the maternal—relative to the fetal—side of the placenta. Serine, alanine, taurine and threonine are all metabolites that readily cross the placenta from the maternal circulation in humans and sheep [6,8]. Aspartate was not shown previously to readily cross the human placenta [24], but evidence in sheep suggests small rates of transfer across the placenta [6]. In addition, previous metabolomic studies reported the presence of aspartate in the term, human placenta [21,23]. Choline and GPC, the most abundant storage form of choline, were also increased in the maternal placental tissue, consistent with evidence of transport across the placenta from the maternal circulation [25]. In addition, we found that tricarboxylic acid (TCA) cycle intermediates, including citrate, succinate, and acetate, were elevated in the maternal placental tissue. TCA cycle metabolism occurs in the human placenta, as evidenced by the study of placental mitochondria [26,27]. However, it is unknown in both humans and sheep if the TCA cycle intermediates measured in these studies are delivered to the placenta by the mother or produced in the placental tissue. Still, metabolomics studies identified the presence of TCA cycle intermediates in maternal blood prior to delivery, with citrate increasing throughout gestation, suggesting increased transport to the placenta and fetus [28,29].

VLDL and formate were the only detected features that were significantly more abundant in the fetal placental tissue. VLDLs have been found in human cord blood of term infants and are important in lung surfactant stimulation [30,31,32,33]. Formate is important in the placental synthesis of folate, a vitamin involved in DNA replication and required for healthy fetal development [34]. Recent studies in sheep found higher levels of formate in the fetus and amniotic fluid than in the maternal circulation, suggesting production by the placenta or fetus itself [35]. Therefore, the elevation of these metabolites in the fetal placental tissue could indicate their importance in maintaining a healthy pregnancy during late gestation.

In this study, we also measured the stability of amino acids and TCA cycle intermediates in placental tissue up to 24 h following delivery in non-labored women undergoing cesarean delivery. In both maternal and fetal tissues, amino acids, TCA cycle intermediates, choline and choline derivatives, formate, and niacinamide changed in concentration within 1 h following delivery. As discussed above, amino acids, TCA cycle intermediates, and formate have all been shown to be important metabolic substrates for both the placenta and the fetus. Choline is an important substrate in cellular membrane formation and neurodevelopment in the fetus [36,37]. Our data suggest that, similar to other tissues, GPC and, to a lesser extent, PC, are two major storage forms of choline in the placenta. GPC was significantly depleted within 15 min following placental delivery, while choline was significantly elevated within 30 min in the maternal placental tissue. However, PC did not show significant decreases until 24 h following delivery. In addition, glycerol, released from GPC in the production of PC, was significantly elevated in the maternal placental tissue 30 min following delivery. In the fetal tissue, GPC was significantly diminished 1 h following delivery, while choline was significantly elevated. However, in the fetal placental tissue, PC was significantly diminished at 15 min, and glycerol was not significantly elevated until 24 h following delivery. This, along with the evidence of elevated GPC in the maternal placental tissue (Table 1), suggests that the maternal surface has greater choline storage and breakdown than the fetal tissue. Another metabolite found to significantly decrease in both the maternal and the fetal surfaces of the placenta is niacinamide (vitamin B3), important in the synthesis of the cofactor nicotinamide adenine dinucleotide (NAD) for glycolysis and cellular respiration. Although it is not known if niacinamide is produced in the placenta or the fetus, it has been reported in the human placenta tissue and could be an important substrate in regulating the TCA cycle [38].

Many metabolites did not show a significant alteration within 1 h or even 24 h following delivery. In both maternal and fetal tissues, metabolites that did not show changes within 24 h following delivery included amino acids (aspartate, asparagine, lysine, taurine, and serine), ketones (3-HB and acetone), pyrimidine and purine degradation intermediates (uridine and inosine), as well as fumarate and creatine. This could be due to the stability of these metabolites in the placental tissue or the breakdown of other metabolic end products to produce a relatively constant concentration.

The maternal tissue displayed a greater sensitivity to metabolic alterations than the fetal tissue collected from the placenta. This could be explained by the greater abundance of metabolites observed in maternal compared to fetal placental tissue (Table 1), which reflects the fact that many of these metabolites are normally transported from the maternal to the fetal compartment via a concentration gradient. However, even for metabolites that did not significantly differ between maternal and fetal placental specimens, including PC and lactate, there were still significant changes before 1 h following delivery in the fetal placental specimens.

## 4. Materials and Methods

### 4.1. Sample Collection

Thirteen, gravid full-term subjects were identified at the University of Florida Shands Hospital and gave their written, informed consent for participation. The protocol was approved by the Institutional Review Board (IRB) at the University of Florida (UF IRB20150007). The placentas were collected immediately following cesarean delivery, and the tissue specimens were collected from the whole placenta at five time points postdelivery: (1) <5 min (*n* = 6); (2) 15 min (*n* = 13); (3) 30 min (*n* = 13); (4) 1 h (*n* = 13); (5) 24 h (*n* = 13). The tissue specimens were immediately frozen in liquid nitrogen following collection. The whole placentas were kept at 4 °C between sampling time points. Tissue specimens were collected from two areas of the maternal (approximately 1 × 1 cm area; depth of 1 cm) and fetal surfaces (approximately 1 × 1 cm area; depth of 0.3 cm) of each placenta at each time point, resulting in 24 total specimens for the <5 min time point and 52 total specimens for all other time points. For five fetal and one maternal specimen, there was not enough tissue for metabolomic analysis. Therefore, the total number of specimens for NMR data collection at each time point was as follows: (1) <5 min (*n* = 12 fetal, *n* =12 maternal); (2) 15 min (*n* = 24 fetal, *n* = 25 maternal); (3) 30 min (*n* = 25 fetal, *n* = 26 maternal); (4) 1 h (*n* = 25 fetal, *n* = 26 maternal); and (5) 24 h (*n* = 25 fetal, *n* = 26 maternal). The samples were stored at −80 °C until analysis.

### 4.2. Tissue Preparation

The placental tissue samples (24.15–192.21 mg; mean 126.21 mg) were cut on dry ice and washed with ice-cold 0.85% sodium chloride to remove the excess blood before being placed in prechilled tubes. Five hundred μL of ice-cold 50/50 methanol/water was added to each sample before vortexing for 1 min. Homogenization and sonication methods were compared for metabolite extractions. Equivalent results were observed for both methods and, therefore, sonication was used because of its ability for high-throughput sample preparation. The samples were sonicated for 20 min to extract the metabolites and then spun at 14,000 rcf for 15 min at 4 °C. Four hundred and fifty μL of supernatant was transferred to a new microcentrifuge tube and concentrated overnight using a CentriVap Benchtop Vacuum Concentrator (Labconco, Kanas City, MO, USA). The samples were frozen at −80 °C until metabolomics analysis.

### 4.3. Metabolomic Analysis

Nuclear magnetic resonance (NMR) spectroscopy was used to identify and quantify the metabolites in the placental tissue. The concentrated tissue specimens were thawed, reconstituted in 600 μL of 100 mM sodium phosphate buffer at pH 7.0, and vortexed until the pellets dissolved [39]. The samples were centrifuged at 14,000 rcf for 15 min at 4 °C before transferring 590 μL into 5 mm NMR tubes (Bruker Biospin, Billerica, MA, USA). The samples were analyzed on an Avance III HD 600 MHz Bruker NMR spectrometer equipped with a Bruker SampleJet cooled to 5.6 °C. Data were acquired using a one-dimensional (1D) experiment with T_2_ filter using Carr–Purcell–Meiboom–Gill (CPMG) pulse sequence with water presaturation for metabolite quantitation and two-dimensional (2D) ^1^H-^13^C heteronuclear single quantum correlation (HSQC) and HSQC–TOCSY (HSQC–total correlation spectroscopy) for metabolite identification. A total of 38 metabolites were identified using Bruker AssureNMR software (Bruker Biospin, USA) with BBiorefcode metabolite database and COLMARm [40]. The metabolites were assigned a confidence level ranging from 1 to 5, with 5 being the highest. The scale is defined as follows: (1) putatively characterized compound classes or annotated compounds, (2) matched to literature and/or 1D BBiorefcode compound (AssureNMR), (3) matched to HSQC (AssureNMR), (4) matched to HSQC and validated by HSQC–TOCSY (COLMARm), and (5) validated by spiking the authentic compound into sample. The metabolite confidence levels are reported in Table S1. The spectra were processed using Bruker Topspin 3.6 software and in-house MATLAB scripts. All raw and processed data is available on the Metabolomics workbench (http://www.metabolomicsworkbench.org/), along with detailed experimental NMR and analysis methods.

### 4.4. Statistical Analysis

The data were normalized using probabilistic quotient normalization (PQN) and Pareto-scaled, before statistical analysis [41,42]. Multivariate analyses of processed spectra were performed using in-house MATLAB scripts (https://github.com/artedison/Edison_Lab_Shared_Metabolomics_UGA). Orthogonal signal correction partial least squares discriminant analysis (OSC–PLSDA) was conducted using the PLS Toolbox for MATLAB (Eigenvector Research Inc., Manson, WA, USA) to determine metabolites that differed between the maternal and fetal surfaces of the placenta [43]. Partial least squares discriminant analysis (PLS-DA) was used to identify metabolites that differed between time points [44]. The principal component analysis (PCA) scores plots are shown in Figures S1–S3. Univariate statistics were performed on metabolite concentrations of PQN-normalized spectra, and all p-values were subject to false discovery rate using the Benjamini–Hochberg method [45]. A Student’s *t*-test with an FDR-correction was used to determine significant metabolites (FDR-corrected, *p* < 0.05) that differed between the maternal and fetal tissue specimens. A one-way ANOVA was used to determine the metabolites that differed between time points in maternal and fetal specimens (FDR-corrected, *p* < 0.05). A Tukey–Kramer post-hoc analysis was used to determine which time points significantly differed.

## 5. Conclusions

In this study, we show significant metabolic differences between tissue collected from the maternal surface of the human term placenta and that collected from the fetal surface. These results will facilitate future studies that could lead to a better understanding of human placental biology and of how it is disrupted by metabolic diseases such as diabetes or hypertension during pregnancy. In addition, we revealed that these tissue types have distinct profiles of metabolites at the four sampling times following cesarean delivery and that many metabolites remain stable in the fetal placental tissue up to 1 h following cesarean delivery. This challenges previous literature indicating that the immediate processing of placental specimens is necessary to see significant metabolite alterations [23]. Our study is limited, in that we did not separate the maternal and fetal tissues microscopically in order to assure rapid processing at the first time points. Future studies are needed to more completely divide these tissue types to gain a more complete metabolic profile of the human placenta and to evaluate these metabolic changes in placentas from women in labor, as the onset of labor is likely to also induce changes in the placenta metabolome prior to delivery. However, the information presented here reveals that sampling from just the maternal or the fetal surfaces of the placenta could lead to the loss of valuable metabolic information, and future studies should include both maternal and fetal tissue specimens to gain a better understanding of how placental metabolism is altered as a consequence of maternal or fetal complications during pregnancy.

## Figures and Tables

**Table 1:** Metabolites identified on maternal and fetal surfaces of the placenta at 15 min postdelivery.

Metabolite	Mean (SE) ^a^		FDR-Corrected *p*-Value	Fold Change: Maternal vs. Fetal
	Maternal	Fetal		
Choline	3.372 (0.113)	2.142 (0.165)	3.6 × 10^−4^	1.57
Serine	1.917 (0.070)	1.409 (0.052)	4.0 × 10^−4^	1.36
Threonine	0.395 (0.007)	0.349 (0.004)	4.0 × 10^−4^	1.13
Citrate	0.475 (0.019)	0.356 (0.012)	9.9 × 10^−4^	1.34
Glycerol	0.210 (0.008)	0.162 (0.006)	4.3 × 10^−3^	1.29
Acetate	0.335 (0.008)	0.278 (0.010)	5.2 × 10^−3^	1.21
Succinate	0.230 (0.011)	0.174 (0.007)	8.5 × 10^−3^	1.32
Formate	0.037 (0.001)	0.048 (0.002)	9.9 × 10^−3^	0.78
Aspartate	0.152 (0.003)	0.128 (0.006)	0.01	1.19
VLDL ^b^	1.333 (0.049)	1.593 (0.059)	0.03	0.84
Taurine	2.787 (0.099)	2.387 (0.070)	0.04	1.17
Niacinamide	0.040 (0.001)	0.035 (0.001)	0.04	1.16
Alanine	1.171 (0.029)	1.026 (0.037)	0.05	1.14
GPC ^c^	2.769 (0.207)	1.960 (0.160)	0.05	1.41
PC ^d^	0.987 (0.034)	0.847 (0.036)	0.07	1.17
Uridine	0.056 (0.009)	0.031 (0.004)	0.12	1.79
Fumarate	0.009 (0.001)	0.011 (0.001)	0.20	0.79
Glutamine	0.710 (0.018)	0.652 (0.021)	0.20	1.09
Creatine	0.519 (0.027)	0.679 (0.075)	0.21	0.76
Glucose	0.826 (0.033)	0.948 (0.050)	0.21	0.87
Uracil	0.058 (0.002)	0.050 (0.004)	0.24	1.15
3-HB ^e^	0.321 (0.021)	0.392 (0.032)	0.25	0.82
Asparagine	0.251 (0.004)	0.261 (0.003)	0.29	0.96
Myo-inositol	1.520 (0.050)	1.378 (0.068)	0.29	1.10
Glycine	0.653 (0.020)	0.599 (0.030)	0.37	1.09
Inosine	0.034 (0.006)	0.025 (0.002)	0.38	1.37
Glutamate	1.933 (0.049)	1.839 (0.053)	0.40	1.05
Histidine	0.080 (0.002)	0.076 (0.002)	0.40	1.05
Acetone	0.102 (0.004)	0.093 (0.006)	0.41	1.10
Leucine	1.057 (0.022)	0.995 (0.047)	0.43	1.06
Lactate	10.632 (0.411)	10.033 (0.427)	0.52	1.06
Valine	0.467 (0.012)	0.448 (0.015)	0.53	1.04
Tyrosine	0.118 (0.003)	0.123 (0.005)	0.63	0.96
Phenylalanine	0.536 (0.011)	0.548 (0.021)	0.79	0.98
Hypoxanthine	0.268 (0.007)	0.271 (0.009)	0.88	0.99
Glutathione	0.104 (0.005)	0.102 (0.005)	0.88	1.02
Lysine	0.390 (0.008)	0.387 (0.012)	0.90	1.01
Isoleucine	0.232 (0.005)	0.231 (0.007)	0.93	1.00

^a^ Means and standard error (SE) were calculated after normalization; ^b^ VLDL: Very-low-density lipoprotein; ^c^ GPC: Glycerophosphocholine; ^d^ PC: Phosphocholine. ^e^ 3-HB: 3-hydroxybutyrate.

**Table 2:** Metabolites abundance over 24 h on the surface of the maternal placenta.

Metabolite	Mean (SE) ^a^					FDR-Corrected *p*-Value	Fold Change over 1 h
	<5 min	15 min	30 min	1 h	24 h		
Glycerol	0.147 (0.021)	0.210 (0.014)	0.235 (0.014)	0.302 (0.014)	0.514 (0.014)	2.03 × 10^−31^	2.06
Lactate	5.370 (0.750)	10.632 (0.520)	11.876 (0.510)	13.896 (0.510)	19.354 (0.510)	1.57 × 10^−28^	2.59
Choline	2.558 (0.297)	3.372 (0.206)	3.840 (0.202)	4.459 (0.202)	5.845 (0.202)	1.41 × 10^−15^	1.74
GPC ^b^	4.363 (0.318)	2.769 (0.220)	2.763 (0.216)	2.395 (0.216)	1.174 (0.216)	8.25 × 10^−11^	0.55
Glucose	1.031 (0.059)	0.826 (0.041)	0.773 (0.040)	0.741 (0.040)	0.472 (0.040)	8.48 × 10^−11^	0.72
Glycine	0.603 (0.047)	0.653 (0.033)	0.700 (0.032)	0.772 (0.032)	0.958 (0.032)	1.66 × 10^−9^	1.28
PC ^c^	1.109 (0.068)	0.987 (0.047)	1.050 (0.046)	0.934 (0.046)	0.606 (0.046)	1.73 × 10^−9^	0.84
Uracil	0.058 (0.009)	0.058 (0.006)	0.063 (0.006)	0.072 (0.006)	0.112 (0.006)	1.81 × 10^−8^	1.25
Formate	0.046 (0.003)	0.037 (0.002)	0.035 (0.002)	0.030 (0.002)	0.026 (0.002)	1.81 × 10^−8^	0.66
Tyrosine	0.112 (0.009)	0.118 (0.006)	0.127 (0.006)	0.141 (0.006)	0.164 (0.006)	8.43 × 10^−7^	1.26
Phenylalanine	0.506 (0.032)	0.536 (0.022)	0.563 (0.022)	0.615 (0.022)	0.699 (0.022)	1.49 × 10^−6^	1.22
Isoleucine	0.209 (0.013)	0.232 (0.009)	0.244 (0.009)	0.265 (0.009)	0.292 (0.009)	4.20 × 10^−6^	1.27
Leucine	0.925 (0.070)	1.057 (0.048)	1.117 (0.048)	1.237 (0.048)	1.346 (0.048)	1.23 × 10^−5^	1.34
Acetate	0.322 (0.019)	0.335 (0.013)	0.366 (0.013)	0.392 (0.013)	0.423 (0.013)	1.24 × 10^−5^	1.22
Valine	0.441 (0.032)	0.467 (0.022)	0.499 (0.021)	0.544 (0.021)	0.604 (0.021)	6.16 × 10^−5^	1.23
Citrate	0.421 (0.028)	0.475 (0.019)	0.487 (0.019)	0.450 (0.019)	0.360 (0.019)	9.97 × 10^−5^	1.07
Alanine	1.051 (0.081)	1.171 (0.056)	1.229 (0.055)	1.324 (0.055)	1.452 (0.055)	8.05 × 10^−4^	1.26
Glutamine	0.615 (0.029)	0.710 (0.020)	0.739 (0.020)	0.756 (0.020)	0.707 (0.020)	4.71 × 10^−3^	1.23
Threonine	0.381 (0.009)	0.395 (0.006)	0.393 (0.006)	0.388 (0.006)	0.364 (0.006)	0.01	1.02
Glutathione	0.085 (0.009)	0.104 (0.006)	0.114 (0.006)	0.099 (0.006)	0.086 (0.006)	0.01	1.16
Hypoxanthine	0.288 (0.016)	0.268 (0.011)	0.278 (0.011)	0.280 (0.011)	0.319 (0.011)	0.02	0.97
Succinate	0.168 (0.021)	0.230 (0.014)	0.239 (0.014)	0.252 (0.014)	0.248 (0.014)	0.03	1.50
Niacinamide	0.048 (0.002)	0.040 (0.001)	0.039 (0.001)	0.040 (0.001)	0.042 (0.001)	0.03	0.84
VLDL ^d^	1.293 (0.097)	1.333 (0.067)	1.250 (0.066)	1.225 (0.066)	1.033 (0.066)	0.04	0.95
Histidine	0.075 (0.005)	0.080 (0.003)	0.078 (0.003)	0.083 (0.003)	0.068 (0.003)	0.04	1.11
Myo-inositol	1.521 (0.081)	1.520 (0.056)	1.568 (0.055)	1.559 (0.055)	1.342 (0.055)	0.04	1.02
Glutamate	1.820 (0.089)	1.933 (0.062)	2.001 (0.061)	2.123 (0.061)	2.080 (0.061)	<0.05	1.17
Lysine	0.375 (0.023)	0.390 (0.016)	0.407 (0.015)	0.422 (0.015)	0.448 (0.015)	>0.05	1.13
Acetone	0.103 (0.007)	0.102 (0.005)	0.105 (0.005)	0.096 (0.005)	0.087 (0.005)	0.09	0.93
Aspartate	0.137 (0.008)	0.152 (0.006)	0.155 (0.005)	0.160 (0.005)	0.163 (0.005)	0.12	1.17
Inosine	0.028 (0.006)	0.034 (0.004)	0.022 (0.004)	0.021 (0.004)	0.024 (0.004)	0.14	0.72
Serine	1.784 (0.107)	1.917 (0.074)	1.923 (0.073)	1.966 (0.073)	1.736 (0.073)	0.19	1.10
Taurine	2.803 (0.141)	2.787 (0.098)	2.792 (0.096)	2.844 (0.096)	2.549 (0.096)	0.25	1.02
Fumarate	0.008 (0.001)	0.009 (0.001)	0.009 (0.001)	0.009 (0.001)	0.011 (0.001)	0.29	1.11
Asparagine	0.259 (0.008)	0.251 (0.005)	0.259 (0.005)	0.265 (0.005)	0.263 (0.005)	0.44	1.03
Uridine	0.050 (0.010)	0.056 (0.007)	0.040 (0.007)	0.042 (0.007)	0.041 (0.007)	0.44	0.84
Creatine	0.560 (0.043)	0.519 (0.030)	0.500 (0.029)	0.513 (0.029)	0.535 (0.029)	0.82	0.92
3-HB ^e^	0.297 (0.042)	0.321 (0.029)	0.320 (0.029)	0.306 (0.029)	0.318 (0.029)	0.99	1.03

^a^ Means and standard error (SE) were calculated after normalization; ^b^ GPC: Glycerophosphocholine; ^c^ PC: Phosphocholine; ^d^ VLDL: Very-low-density lipoprotein; ^e^ 3-HB: 3-hydroxybutyrate.

**Table 3:** Metabolites abundance over 24 h on the surface of the fetal placenta.

Metabolite	Mean (SE) ^a^					FDR-Corrected *p*-Value	Fold Change over 1 h
	<5 min	15 min	30 min	1 h	24 h		
Glycerol	0.140 (0.019)	0.162 (0.013)	0.184 (0.013)	0.193 (0.013)	0.381 (0.013)	4.4 × 10^−22^	1.38
Lactate	7.824 (1.109)	10.033 (0.784)	12.127 (0.768)	14.392 (0.768)	22.069 (0.768)	3.4 × 10^−20^	1.84
Choline	1.730 (0.282)	2.142 (0.200)	2.594 (0.196)	2.683 (0.196)	4.158 (0.196)	1.5 × 10^−10^	1.55
PC ^b^	1.089 (0.069)	0.847 (0.049)	0.886 (0.048)	0.891 (0.048)	0.589 (0.048)	2.6 × 10^−6^	0.82
GPC ^c^	2.443 (0.221)	1.960 (0.157)	1.949 (0.153)	1.640 (0.153)	0.980 (0.153)	7.7 × 10^−6^	0.67
Succcinate	0.175 (0.016)	0.174 (0.012)	0.190 (0.011)	0.218 (0.011)	0.259 (0.011)	2.3 × 10^−5^	1.24
Threonine	0.346 (0.009)	0.349 (0.006)	0.354 (0.006)	0.348 (0.006)	0.311 (0.006)	1.2 × 10^−4^	1.00
Citrate	0.363 (0.021)	0.356 (0.015)	0.380 (0.014)	0.389 (0.014)	0.296 (0.014)	5.9 × 10^−4^	1.07
Uracil	0.052 (0.006)	0.050 (0.004)	0.049 (0.004)	0.049 (0.004)	0.071 (0.004)	4.4 × 10^−3^	0.94
Fumarate	0.010 (0.001)	0.011 (0.001)	0.011 (0.001)	0.012 (0.001)	0.016 (0.001)	6.3 × 10^−3^	1.20
Glycine	0.605 (0.042)	0.599 (0.030)	0.616 (0.029)	0.607 (0.029)	0.735 (0.029)	0.02	1.00
Glutathione	0.092 (0.008)	0.102 (0.006)	0.100 (0.006)	0.107 (0.006)	0.080 (0.006)	0.04	1.17
Niacinamide	0.040 (0.002)	0.035 (0.001)	0.033 (0.001)	0.034 (0.001)	0.037 (0.001)	0.04	0.83
Asparagine	0.255 (0.010)	0.261 (0.007)	0.253 (0.007)	0.251 (0.007)	0.232 (0.007)	0.11	0.98
Acetone	0.101 (0.012)	0.093 (0.009)	0.101 (0.009)	0.094 (0.009)	0.067 (0.009)	0.12	0.94
Glucose	1.140 (0.133)	0.948 (0.094)	1.022 (0.092)	0.770 (0.092)	0.751 (0.092)	0.12	0.68
VLDL ^d^	1.527 (0.114)	1.593 (0.081)	1.528 (0.079)	1.491 (0.079)	1.279 (0.079)	0.16	0.98
Glutamate	1.949 (0.100)	1.839 (0.071)	1.924 (0.069)	1.920 (0.069)	1.693 (0.069)	0.19	0.98
Taurine	2.638 (0.136)	2.387 (0.096)	2.478 (0.094)	2.271 (0.094)	2.269 (0.094)	0.25	0.86
Isoleucine	0.216 (0.010)	0.231 (0.007)	0.226 (0.007)	0.229 (0.007)	0.244 (0.007)	0.29	1.06
Alanine	1.052 (0.061)	1.026 (0.043)	1.030 (0.042)	1.044 (0.042)	1.156 (0.042)	0.29	0.99
Creatine	0.997 (0.126)	0.679 (0.089)	0.620 (0.087)	0.700 (0.087)	0.776 (0.087)	0.29	0.70
Hypoxanthine	0.294 (0.016)	0.271 (0.011)	0.248 (0.011)	0.272 (0.011)	0.274 (0.011)	0.29	0.92
Phenylalanine	0.535 (0.025)	0.548 (0.018)	0.511 (0.018)	0.520 (0.018)	0.563 (0.018)	0.37	0.97
Formate	0.053 (0.006)	0.048 (0.004)	0.047 (0.004)	0.054 (0.004)	0.043 (0.004)	0.51	1.03
Tyrosine	0.124 (0.007)	0.123 (0.005)	0.117 (0.005)	0.117 (0.005)	0.129 (0.005)	0.60	0.94
Glutamine	0.683 (0.033)	0.652 (0.023)	0.687 (0.023)	0.707 (0.023)	0.662 (0.023)	0.67	1.03
Lysine	0.379 (0.019)	0.387 (0.013)	0.360 (0.013)	0.358 (0.013)	0.369 (0.013)	0.67	0.95
Uridine	0.025 (0.008)	0.031 (0.006)	0.040 (0.006)	0.035 (0.006)	0.031 (0.006)	0.75	1.43
Valine	0.451 (0.024)	0.448 (0.017)	0.449 (0.017)	0.444 (0.017)	0.476 (0.017)	0.80	0.98
Serine	1.484 (0.100)	1.409 (0.071)	1.490 (0.069)	1.359 (0.069)	1.458 (0.069)	0.80	0.92
Inosine	0.022 (0.007)	0.025 (0.005)	0.033 (0.005)	0.029 (0.005)	0.031 (0.005)	0.80	1.33
Histidine	0.076 (0.004)	0.076 (0.003)	0.074 (0.003)	0.073 (0.003)	0.071 (0.003)	0.87	0.97
Leucine	0.937 (0.057)	0.995 (0.040)	0.950 (0.039)	0.944 (0.039)	0.991 (0.039)	0.88	1.01
3-HB ^e^	0.417 (0.066)	0.392 (0.047)	0.432 (0.046)	0.438 (0.046)	0.402 (0.046)	0.95	1.05
Acetate	0.271 (0.016)	0.278 (0.012)	0.287 (0.011)	0.284 (0.011)	0.284 (0.011)	0.95	1.05
Aspartate	0.122 (0.009)	0.128 (0.006)	0.128 (0.006)	0.127 (0.006)	0.123 (0.006)	0.95	1.04
Myo-inositol	1.363 (0.144)	1.378 (0.102)	1.475 (0.100)	1.466 (0.100)	1.429 (0.100)	0.95	1.08

^a^ Means and standard error (SE) were calculated after normalization; ^b^ PC: Phosphocholine; ^c^ GPC: Glycerophosphocholine; ^d^ VLDL: Very-low-density lipoprotein; ^e^ 3-HB: 3-hydroxybutyrate.

## Supplementary Materials

The following are available online at http://www.mdpi.com/2218-1989/8/1/10/s1. The following are in the attached Supplementary Material document: Figures S1–S5, Tables S1–S3.
