# Supplementary material for: Global Metabolomics of the Placenta Reveals Distinct Metabolic Profiles between Maternal and Fetal Placental Tissues Following Delivery in Non-Labored Women
Source: Metabolites. 2018 Jan 23;8(1):10. doi: 10.3390/metabo8010010 (PMC5876000; doi:10.3390/metabo8010010)
Supplement: Supplementary file 1 [file metabolites-08-00010-s001.pdf]

Global Metabolomics of the Placenta Reveals Distinct Metabolic Profiles Between Maternal and Fetal Placental Tissue Following Delivery in Non-Labored Women

Jacquelyn Walejko, BS[1]; Anushka Chelliah, MD[2]; Maureen Keller-Wood, PhD[3]; Anthony Gregg, MD, MBA[4]; Arthur S. Edison, PhD[5]

[1] Department of Biochemistry & Molecular Biology, University of Florida, Gainesville, FL, US; jwalejko@ufl.edu
[2] Department of Obstetrics, Gynecology, and Reproductive Sciences, University of Texas Health Science Center at Houston, UT Health, Houston, TX, 77030 US; anushka.chelliah@uth.tmc.edu
[3] Department of Pharmacodynamics, University of Florida, Gainesville, FL, 32610 US; kellerwd@cop.ufl.edu
[4] Department of Obstetrics and Gynecology, University of Florida, Gainesville, FL, 32610 US; greggar@ufl.edu
[5] Departments of Genetics and Biochemistry & Molecular Biology, Complex Carbohydrate Research Center, University of Georgia, Athens, GA, 30602 US; aedison@uga.edu
* Correspondence: aedison@uga.edu; Tel.: +1-706-542-8156

Supplementary Material

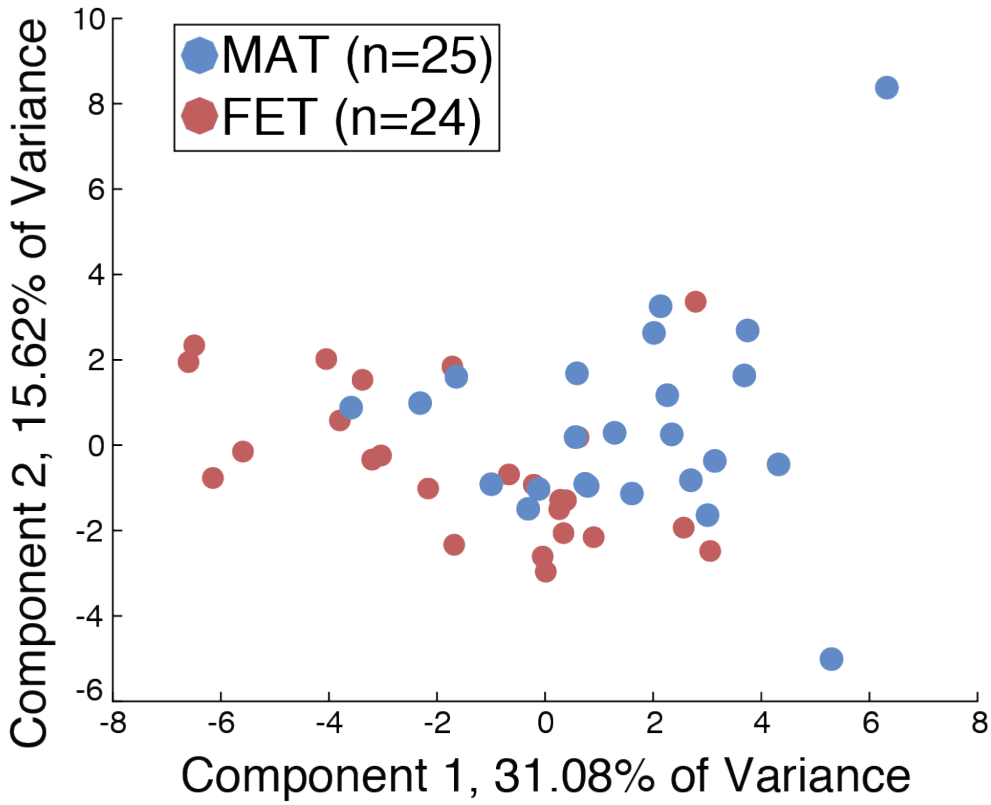

Figure S1: PCA scores plot of maternal and fetal spectra from 13 placentas at 15 min postdelivery. Maternal (MAT, n=25) samples are displayed in blue while fetal (FET, n=24) are displayed in red.

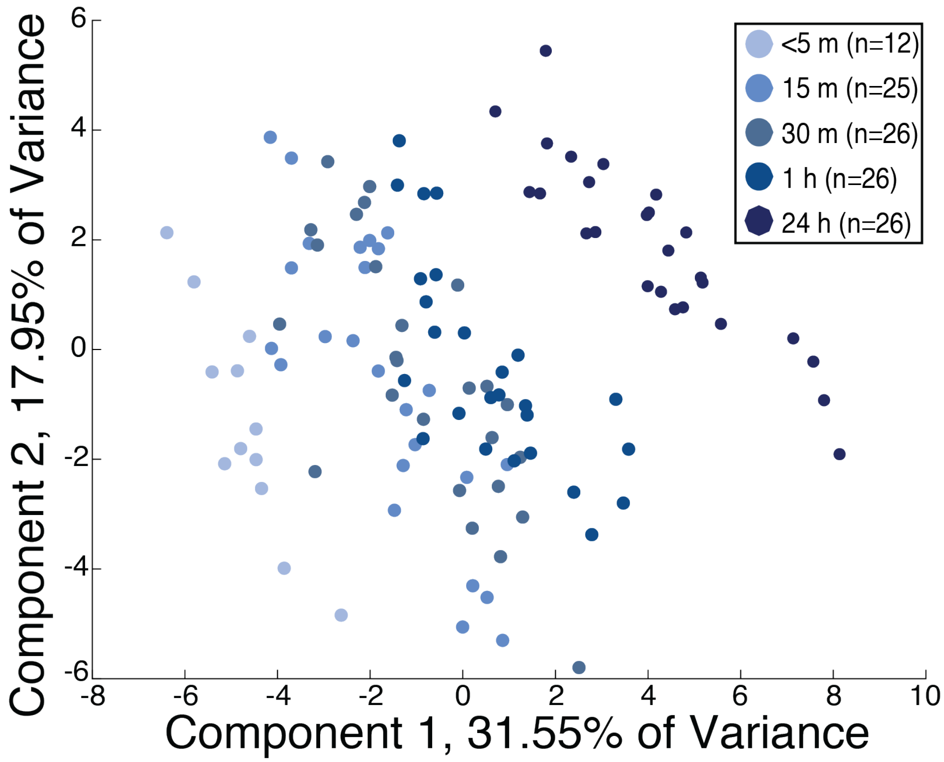

Figure S2 PCA scores plot of specimens from the maternal surface of 13 placentas at 5 time points postdelivery: **1)** <5 min (n=12), **2)** 15 min (n=25), **3)** 30 min (n=26), **4)** 1 h (n=26), and **5)** 24 h (n=26). Time points are represented as varying shades of blue from light (<5 min) to dark (24 h).

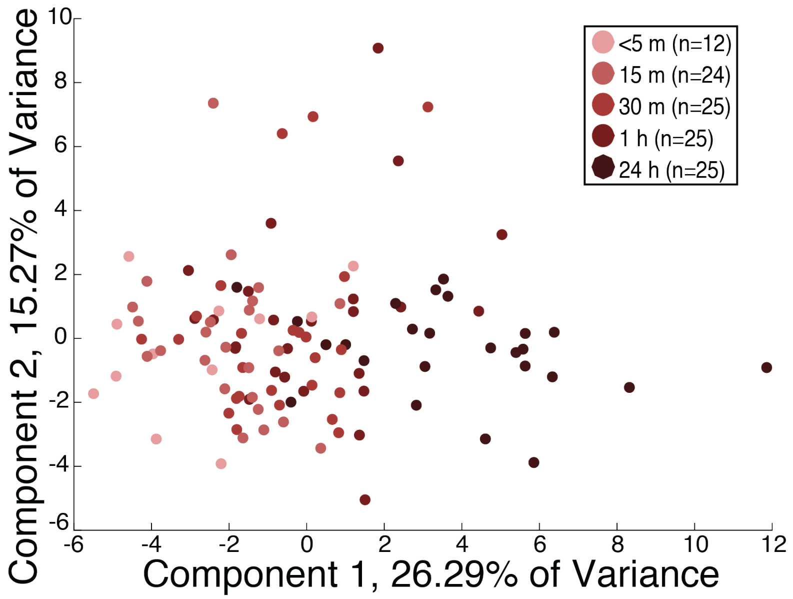

Figure S3: PCA scores plot of specimens from the fetal surface of the placenta from 13 placentas at 5 time points postdelivery: **1)** <5 min (n=12), **2)** 15 min (n=24), **3)** 30 min (n=25), **4)** 1 h (n=25), and **5)** 24 h (n=25). Time points are represented as varying shades of red from light (<5 min) to dark (24 h).

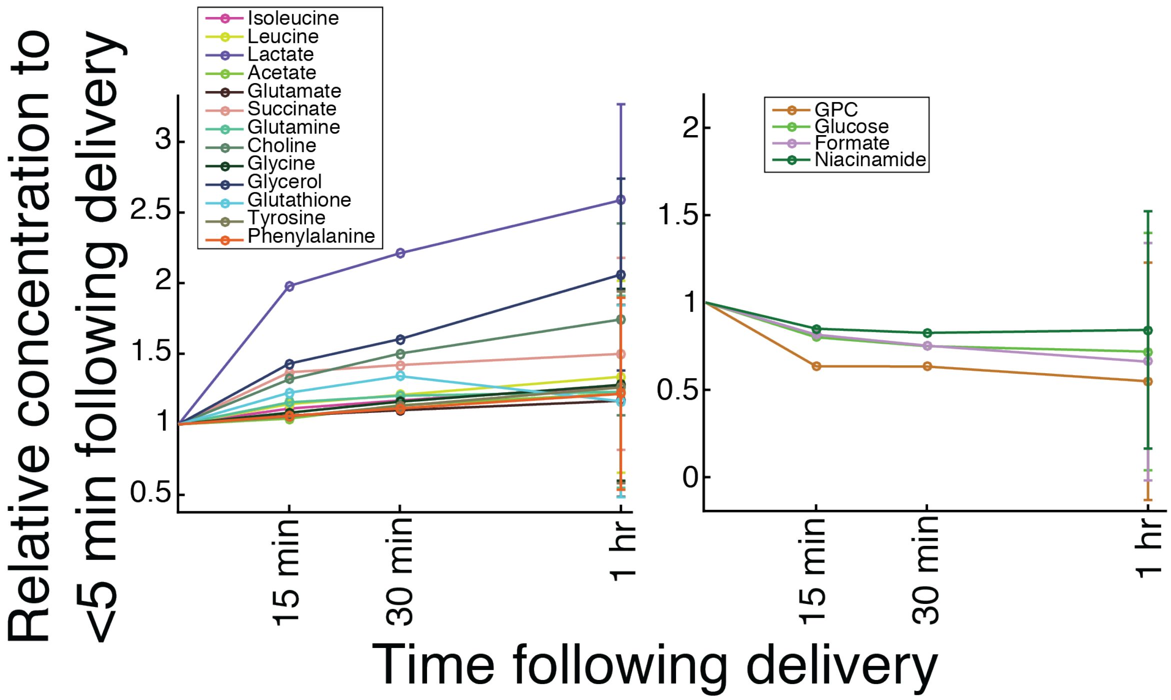

Figure S4. (A) Relative concentration of metabolites on the maternal surface of the placenta that are significantly increased (FDR-corrected p<0.05) prior to 1 h following delivery. **(B)** Glucose, GPC, formate, and niacinamide decrease linearly prior to 1 h following delivery, with one or more time points significantly decreased (FDR-corrected p<0.05) over prior to 1 h following delivery. Concentrations are normalized to the concentration <5 min following delivery for each metabolite. Standard errors are shown on the 1 h datapoint for each metabolite. Full post-hoc analysis is shown in Table S2.

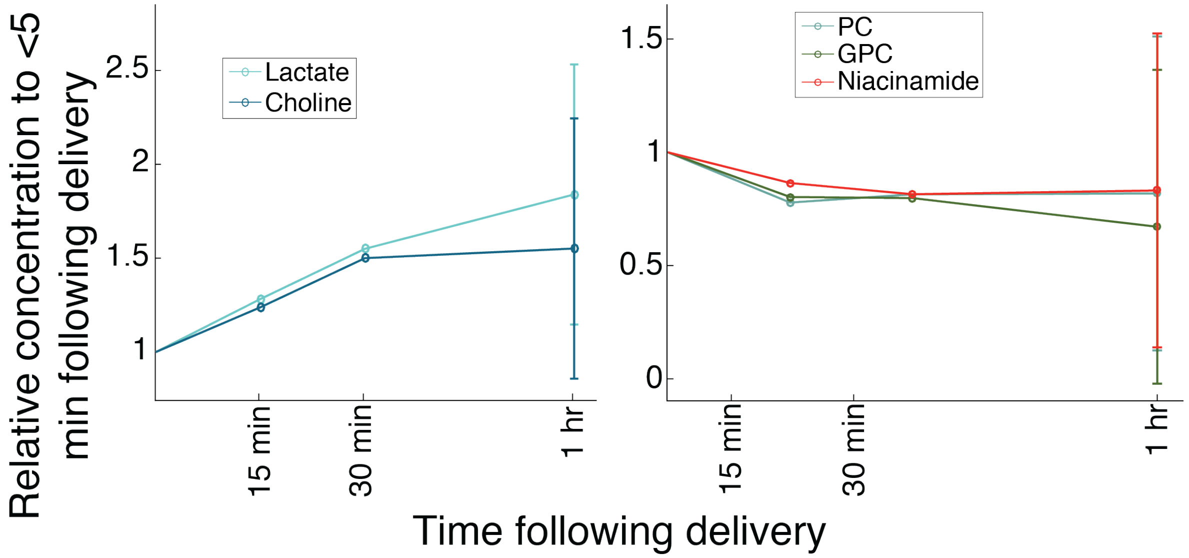

Figure S5. (A) Unlike the maternal surfaces of the placenta, lactate and choline are only metabolites significantly increased (FDR-corrected p<0.05) prior to 1 h following delivery on the fetal surface of the placenta. **(B)** PC, GPC, and niacinamide decrease linearly prior to 1 h following delivery, with one or more time points significantly decreased (FDR-corrected p<0.05) over prior to 1 h following delivery.

Concentrations are normalized to the concentration <5 min following delivery for each metabolite. Standard errors are shown on the 1 h data-point for each metabolite. Full post-hoc analysis is shown in Table S3.

Table S1. Placental Metabolites and Confidence Levels

**Metabolite**	**Chemical shift range (ppm)**		**Confidence level**[a]
VLDL[b]	0.80	0.90	2
Isoleucine	0.99	1.02	3
Leucine	0.94	0.97	4
Valine	1.02	1.05	4
3-HB[c]	1.18	1.20	2
Lactate	1.30	1.34	4
Alanine	1.46	1.49	4
Acetate	1.91	1.92	3
Acetone	2.22	2.23	2
Glutamate	2.32	2.37	4
Succinate	2.40	2.41	3
Glutamine	2.43	2.47	4
Citrate	2.53	2.57	2
Aspartate	2.81	2.83	4
Asparagine	2.83	2.88	4
Lysine	3.00	3.02	4
Creatine	3.02	3.04	4
Choline	3.19	3.20	4
PC[c]	3.21	3.22	4
GPC[e]	3.22	3.23	4
Taurine	3.40	3.44	4
Glucose	3.44	3.48	4
Myo-inositol	3.59	3.62	4
Glycine	3.55	3.55	3
Glycerol	3.56	3.57	4
Serine	3.83	3.85	4
Threonine	4.23	4.26	4
Glutathione	4.55	4.58	2
Uracil	5.79	5.81	4
Uridine	5.88	5.92	2
Fumarate	6.51	6.51	2
Tyrosine	6.88	6.91	4
Phenylalanine	7.30	7.44	4

Histidine	8.15	8.17	3
Hypoxanthine	8.18	8.21	3
Inosine	8.22	8.23	2
Formate	8.44	8.45	2
Niacinamide	8.70	8.71	3

[a]Confidence scale is defined as follows: 1) putatively characterized compound classes or annotated compounds, 2) matched to literature and/or 1D SBASE compound (AssureNMR), 3) matched to HSQC (AssureNMR), 4) matched to HSQC and validated by HSQC-TOCSY (COLMARm), and 5) validated by spiking the authentic compound into sample
[b]VLDL: Very low-density lipoprotein
[c]3-HB: 3-hydroxybutyrate
[d]PC: Phosphocholine
[e]GPC: Glycerophosphocholine

Table S2. Tukey-Kramer Post-Hoc Results for Maternal Surface of Placenta

VLDL					
Group 1	Group 2	Lower CI	Estimate	Upper CI	p-value
<5m	15m	-0.37	-0.04	0.29	1.00
<5m	30m	-0.28	0.04	0.37	1.00
<5m	1h	-0.26	0.07	0.39	0.98
<5m	24h	-0.06	0.26	0.58	0.18
15m	30m	-0.18	0.08	0.34	0.90
15m	1h	-0.15	0.11	0.37	0.78
15m	24h	0.04	0.30	0.56	0.02
30m	1h	-0.23	0.02	0.28	1.00
30m	24h	-0.04	0.22	0.47	0.14
1h	24h	-0.07	0.19	0.45	0.24
Isoleucine					
<5m	15m	-0.07	-0.02	0.02	0.61
<5m	30m	-0.08	-0.04	0.01	0.19
<5m	1h	-0.10	-0.06	-0.01	0.01
<5m	24h	-0.13	-0.08	-0.04	9.9E-06
15m	30m	-0.05	-0.01	0.02	0.89
15m	1h	-0.07	-0.03	2.6E-03	0.08
15m	24h	-0.10	-0.06	-0.02	8.7E-05
30m	1h	-0.06	-0.02	0.01	0.46
30m	24h	-0.08	-0.05	-0.01	2.4E-03
1h	24h	-0.06	-0.03	0.01	0.23

Leucine					
<5m	15m	-0.37	-0.13	0.10	0.53
<5m	30m	-0.43	-0.19	0.04	0.16
<5m	1h	-0.55	-0.31	-0.08	3.2E-03
<5m	24h	-0.66	-0.42	-0.19	2.3E-05
15m	30m	-0.25	-0.06	0.13	0.90
15m	1h	-0.37	-0.18	0.01	0.07
15m	24h	-0.48	-0.29	-0.10	4.1E-04
30m	1h	-0.31	-0.12	0.07	0.39
30m	24h	-0.42	-0.23	-0.04	0.01
1h	24h	-0.30	-0.11	0.08	0.48
Valine					
<5m	15m	-0.13	-0.03	0.08	0.96
<5m	30m	-0.16	-0.06	0.05	0.56
<5m	1h	-0.21	-0.10	3.2E-03	0.06
<5m	24h	-0.27	-0.16	-0.06	4.0E-04
15m	30m	-0.12	-0.03	0.05	0.83
15m	1h	-0.16	-0.08	0.01	0.09
15m	24h	-0.22	-0.14	-0.05	1.7E-04
30m	1h	-0.13	-0.04	0.04	0.58
30m	24h	-0.19	-0.11	-0.02	0.01
1h	24h	-0.14	-0.06	0.02	0.28
3-HB					
<5m	15m	-0.17	-0.02	0.12	0.99
<5m	30m	-0.16	-0.02	0.12	0.99
<5m	1h	-0.15	-0.01	0.13	1.00
<5m	24h	-0.16	-0.02	0.12	0.99
15m	30m	-0.11	1.1E-03	0.11	1.00
15m	1h	-0.10	0.01	0.13	1.00
15m	24h	-0.11	3.0E-03	0.12	1.00
30m	1h	-0.10	0.01	0.13	1.00
30m	24h	-0.11	1.8E-03	0.11	1.00
1h	24h	-0.12	-0.01	0.10	1.00
Lactate					
<5m	15m	-7.79	-5.26	-2.73	7.5E-07
<5m	30m	-9.02	-6.51	-3.99	1.1E-08

<5m	1h	-11.04	-8.53	-6.01	9.9E-09
<5m	24h	-16.50	-13.98	-11.47	9.9E-09
15m	30m	-3.26	-1.24	0.77	0.43
15m	1h	-5.28	-3.26	-1.25	1.7E-04
15m	24h	-10.74	-8.72	-6.70	9.9E-09
30m	1h	-4.02	-2.02	-0.02	0.05
30m	24h	-9.48	-7.48	-5.48	9.9E-09
1h	24h	-7.46	-5.46	-3.46	1.0E-08
Alanine					
<5m	15m	-0.39	-0.12	0.15	0.74
<5m	30m	-0.45	-0.18	0.09	0.37
<5m	1h	-0.55	-0.27	2.9E-04	0.05
<5m	24h	-0.67	-0.40	-0.13	8.3E-04
15m	30m	-0.28	-0.06	0.16	0.95
15m	1h	-0.37	-0.15	0.07	0.31
15m	24h	-0.50	-0.28	-0.06	0.01
30m	1h	-0.31	-0.09	0.12	0.75
30m	24h	-0.44	-0.22	-0.01	0.04
1h	24h	-0.34	-0.13	0.09	0.48
Acetate					
<5m	15m	-0.08	-0.01	0.05	0.98
<5m	30m	-0.11	-0.04	0.02	0.31
<5m	1h	-0.13	-0.07	-0.01	0.02
<5m	24h	-0.16	-0.10	-0.04	1.7E-04
15m	30m	-0.08	-0.03	0.02	0.45
15m	1h	-0.11	-0.06	-0.01	0.02
15m	24h	-0.14	-0.09	-0.04	3.8E-05
30m	1h	-0.08	-0.03	0.02	0.58
30m	24h	-0.11	-0.06	-0.01	0.01
1h	24h	-0.08	-0.03	0.02	0.42
Acetone					
<5m	15m	-0.02	5.4E-04	0.02	1.00
<5m	30m	-0.02	-1.8E-03	0.02	1.00
<5m	1h	-0.02	0.01	0.03	0.91
<5m	24h	-0.01	0.02	0.04	0.32
15m	30m	-0.02	-2.4E-03	0.02	1.00

15m	1h	-0.01	0.01	0.03	0.86
15m	24h	-3.2E-03	0.02	0.03	0.15
30m	1h	-0.01	0.01	0.03	0.66
30m	24h	-6.6E-04	0.02	0.04	0.06
1h	24h	-0.01	0.01	0.03	0.68
Glutamate					
<5m	15m	-0.42	-0.11	0.19	0.83
<5m	30m	-0.48	-0.18	0.12	0.45
<5m	1h	-0.60	-0.30	-3.8E-03	0.05
<5m	24h	-0.56	-0.26	0.04	0.12
15m	30m	-0.31	-0.07	0.17	0.94
15m	1h	-0.43	-0.19	0.05	0.19
15m	24h	-0.39	-0.15	0.09	0.44
30m	1h	-0.36	-0.12	0.12	0.62
30m	24h	-0.32	-0.08	0.16	0.89
1h	24h	-0.20	0.04	0.28	0.99
Succinate					
<5m	15m	-0.13	-0.06	0.01	0.11
<5m	30m	-0.14	-0.07	-6.1E-04	0.05
<5m	1h	-0.15	-0.08	-0.01	0.01
<5m	24h	-0.15	-0.08	-0.01	0.02
15m	30m	-0.07	-0.01	0.05	0.99
15m	1h	-0.08	-0.02	0.03	0.81
15m	24h	-0.07	-0.02	0.04	0.91
30m	1h	-0.07	-0.01	0.04	0.96
30m	24h	-0.06	-0.01	0.05	0.99
1h	24h	-0.05	4.3E-03	0.06	1.00
Glutamine					
<5m	15m	-0.19	-0.10	2.7E-03	0.06
<5m	30m	-0.22	-0.12	-0.03	0.01
<5m	1h	-0.24	-0.14	-0.04	1.0E-03
<5m	24h	-0.19	-0.09	0.01	0.08
15m	30m	-0.11	-0.03	0.05	0.85
15m	1h	-0.12	-0.05	0.03	0.49
15m	24h	-0.07	3.7E-03	0.08	1.00
30m	1h	-0.09	-0.02	0.06	0.97

30m	24h	-0.05	0.03	0.11	0.78
1h	24h	-0.03	0.05	0.13	0.40
Citrate					
<5m	15m	-0.15	-0.05	0.04	0.50
<5m	30m	-0.16	-0.07	0.03	0.29
<5m	1h	-0.12	-0.03	0.06	0.91
<5m	24h	-0.03	0.06	0.15	0.37
15m	30m	-0.09	-0.01	0.06	0.99
15m	1h	-0.05	0.02	0.10	0.89
15m	24h	0.04	0.11	0.19	4.0E-04
30m	1h	-0.04	0.04	0.11	0.64
30m	24h	0.05	0.13	0.20	5.7E-05
1h	24h	0.02	0.09	0.16	0.01
Aspartate					
<5m	15m	-0.04	-0.01	0.01	0.56
<5m	30m	-0.04	-0.02	0.01	0.39
<5m	1h	-0.05	-0.02	4.1E-03	0.14
<5m	24h	-0.05	-0.03	1.3E-03	0.07
15m	30m	-0.02	0.00	0.02	1.00
15m	1h	-0.03	-0.01	0.01	0.83
15m	24h	-0.03	-0.01	0.01	0.63
30m	1h	-0.03	-0.01	0.02	0.95
30m	24h	-0.03	-0.01	0.01	0.82
1h	24h	-0.02	-2.7E-03	0.02	1.00
Asparagine					
<5m	15m	-0.02	0.01	0.03	0.94
<5m	30m	-0.03	1.6E-04	0.03	1.00
<5m	1h	-0.03	-0.01	0.02	0.96
<5m	24h	-0.03	-4.5E-03	0.02	0.99
15m	30m	-0.03	-0.01	0.01	0.88
15m	1h	-0.04	-0.01	0.01	0.36
15m	24h	-0.03	-0.01	0.01	0.53
30m	1h	-0.03	-0.01	0.01	0.90
30m	24h	-0.03	-4.6E-03	0.02	0.97
1h	24h	-0.02	2.1E-03	0.02	1.00

Lysine					
<5m	15m	-0.09	-0.02	0.06	0.98
<5m	30m	-0.11	-0.03	0.04	0.77
<5m	1h	-0.12	-0.05	0.03	0.44
<5m	24h	-0.15	-0.07	3.1E-03	0.07
15m	30m	-0.08	-0.02	0.04	0.94
15m	1h	-0.09	-0.03	0.03	0.61
15m	24h	-0.12	-0.06	3.4E-03	0.07
30m	1h	-0.08	-0.01	0.05	0.96
30m	24h	-0.10	-0.04	0.02	0.34
1h	24h	-0.09	-0.03	0.03	0.75
Creatine					
<5m	15m	-0.10	0.04	0.18	0.93
<5m	30m	-0.08	0.06	0.20	0.78
<5m	1h	-0.10	0.05	0.19	0.89
<5m	24h	-0.12	0.03	0.17	0.99
15m	30m	-0.10	0.02	0.13	0.99
15m	1h	-0.11	0.01	0.12	1.00
15m	24h	-0.13	-0.02	0.10	1.00
30m	1h	-0.13	-0.01	0.10	1.00
30m	24h	-0.15	-0.03	0.08	0.92
1h	24h	-0.13	-0.02	0.09	0.99
Choline					
<5m	15m	-1.82	-0.81	0.19	0.17
<5m	30m	-2.28	-1.28	-0.29	4.7E-03
<5m	1h	-2.90	-1.90	-0.91	6.1E-06
<5m	24h	-4.28	-3.29	-2.29	9.9E-09
15m	30m	-1.27	-0.47	0.33	0.49
15m	1h	-1.89	-1.09	-0.29	2.4E-03
15m	24h	-3.27	-2.47	-1.67	9.9E-09
30m	1h	-1.41	-0.62	0.17	0.20
30m	24h	-2.80	-2.01	-1.21	1.2E-08
1h	24h	-2.18	-1.39	-0.60	3.8E-05
PC					
<5m	15m	-0.11	0.12	0.35	0.59
<5m	30m	-0.17	0.06	0.29	0.95

<5m	1h	-0.05	0.17	0.40	0.22
<5m	24h	0.27	0.50	0.73	1.9E-07
15m	30m	-0.25	-0.06	0.12	0.88
15m	1h	-0.13	0.05	0.24	0.93
15m	24h	0.20	0.38	0.56	8.7E-07
30m	1h	-0.07	0.12	0.30	0.40
30m	24h	0.26	0.44	0.63	1.7E-08
1h	24h	0.15	0.33	0.51	2.3E-05
GPC					
<5m	15m	0.52	1.59	2.67	7.0E-04
<5m	30m	0.53	1.60	2.67	6.0E-04
<5m	1h	0.90	1.97	3.03	1.3E-05
<5m	24h	2.12	3.19	4.26	9.9E-09
15m	30m	-0.85	0.01	0.86	1.00
15m	1h	-0.48	0.37	1.23	0.75
15m	24h	0.74	1.59	2.45	1.1E-05
30m	1h	-0.48	0.37	1.22	0.75
30m	24h	0.74	1.59	2.44	9.2E-06
1h	24h	0.37	1.22	2.07	1.1E-03
Taurine					
<5m	15m	-0.46	0.02	0.49	1.00
<5m	30m	-0.46	0.01	0.48	1.00
<5m	1h	-0.52	-0.04	0.43	1.00
<5m	24h	-0.22	0.25	0.73	0.57
15m	30m	-0.39	-4.9E-03	0.38	1.00
15m	1h	-0.44	-0.06	0.32	0.99
15m	24h	-0.14	0.24	0.62	0.42
30m	1h	-0.43	-0.05	0.32	1.00
30m	24h	-0.13	0.24	0.62	0.38
1h	24h	-0.08	0.30	0.67	0.20
Glucose					
<5m	15m	0.01	0.21	0.40	0.04
<5m	30m	0.06	0.26	0.46	4.0E-03
<5m	1h	0.09	0.29	0.49	8.3E-04
<5m	24h	0.36	0.56	0.76	1.0E-08
15m	30m	-0.11	0.05	0.21	0.89

15m	1h	-0.07	0.08	0.24	0.58
15m	24h	0.19	0.35	0.51	1.2E-07
30m	1h	-0.12	0.03	0.19	0.98
30m	24h	0.14	0.30	0.46	5.5E-06
1h	24h	0.11	0.27	0.43	6.0E-05
Myo-inositol					
<5m	15m	-0.27	9.3E-04	0.27	1.00
<5m	30m	-0.32	-0.05	0.22	0.99
<5m	1h	-0.31	-0.04	0.23	1.00
<5m	24h	-0.09	0.18	0.45	0.36
15m	30m	-0.26	-0.05	0.17	0.97
15m	1h	-0.26	-0.04	0.18	0.99
15m	24h	-0.04	0.18	0.40	0.16
30m	1h	-0.21	0.01	0.22	1.00
30m	24h	0.01	0.23	0.44	0.03
1h	24h	2.5E-03	0.22	0.43	0.05
Glycine					
<5m	15m	-0.21	-0.05	0.11	0.91
<5m	30m	-0.25	-0.10	0.06	0.44
<5m	1h	-0.33	-0.17	-0.01	0.03
<5m	24h	-0.51	-0.35	-0.20	9.6E-08
15m	30m	-0.17	-0.05	0.08	0.84
15m	1h	-0.25	-0.12	0.01	0.08
15m	24h	-0.43	-0.30	-0.18	2.0E-08
30m	1h	-0.20	-0.07	0.05	0.50
30m	24h	-0.38	-0.26	-0.13	1.0E-06
1h	24h	-0.31	-0.19	-0.06	7.4E-04
Glycerol					
<5m	15m	-0.13	-0.06	0.01	0.10
<5m	30m	-0.16	-0.09	-0.02	0.01
<5m	1h	-0.23	-0.16	-0.09	1.2E-07
<5m	24h	-0.44	-0.37	-0.30	9.9E-09
15m	30m	-0.08	-0.03	0.03	0.71
15m	1h	-0.15	-0.09	-0.04	1.2E-04
15m	24h	-0.36	-0.30	-0.25	9.9E-09
30m	1h	-0.12	-0.07	-0.01	0.01

30m	24h	-0.33	-0.28	-0.22	9.9E-09
1h	24h	-0.27	-0.21	-0.16	9.9E-09
Serine					
<5m	15m	-0.49	-0.13	0.23	0.85
<5m	30m	-0.50	-0.14	0.22	0.82
<5m	1h	-0.54	-0.18	0.18	0.62
<5m	24h	-0.31	0.05	0.41	1.00
15m	30m	-0.29	-0.01	0.28	1.00
15m	1h	-0.34	-0.05	0.24	0.99
15m	24h	-0.11	0.18	0.47	0.41
30m	1h	-0.33	-0.04	0.24	0.99
30m	24h	-0.10	0.19	0.47	0.36
1h	24h	-0.05	0.23	0.52	0.17
Threonine					
<5m	15m	-0.05	-0.01	0.02	0.68
<5m	30m	-0.04	-0.01	0.02	0.81
<5m	1h	-0.04	-0.01	0.02	0.96
<5m	24h	-0.01	0.02	0.05	0.58
15m	30m	-0.02	2.5E-03	0.03	1.00
15m	1h	-0.02	0.01	0.03	0.93
15m	24h	0.01	0.03	0.06	0.01
30m	1h	-0.02	4.6E-03	0.03	0.99
30m	24h	4.1E-03	0.03	0.05	0.01
1h	24h	-4.4E-04	0.02	0.05	0.06
Glutathione					
<5m	15m	-0.05	-0.02	0.01	0.36
<5m	30m	-0.06	-0.03	-3.4E-04	0.05
<5m	1h	-0.04	-0.01	0.01	0.67
<5m	24h	-0.03	-1.0E-03	0.03	1.00
15m	30m	-0.03	-0.01	0.01	0.75
15m	1h	-0.02	0.01	0.03	0.97
15m	24h	0.00	0.02	0.04	0.20
30m	1h	-0.01	0.02	0.04	0.36
30m	24h	0.01	0.03	0.05	0.01
1h	24h	-0.01	0.01	0.04	0.52

Uracil					
<5m	15m	-0.03	-2.0E-04	0.03	1.00
<5m	30m	-0.03	-0.01	0.02	0.99
<5m	1h	-0.04	-0.01	0.01	0.64
<5m	24h	-0.08	-0.05	-0.02	1.2E-05
15m	30m	-0.03	-5.0E-03	0.02	0.98
15m	1h	-0.04	-0.01	0.01	0.44
15m	24h	-0.08	-0.05	-0.03	5.6E-08
30m	1h	-0.03	-0.01	0.01	0.79
30m	24h	-0.07	-0.05	-0.03	5.6E-07
1h	24h	-0.06	-0.04	-0.02	7.2E-05
Uridine					
<5m	15m	-0.04	-0.01	0.03	0.99
<5m	30m	-0.02	0.01	0.04	0.91
<5m	1h	-0.03	0.01	0.04	0.96
<5m	24h	-0.02	0.01	0.04	0.94
15m	30m	-0.01	0.02	0.04	0.45
15m	1h	-0.01	0.01	0.04	0.61
15m	24h	-0.01	0.02	0.04	0.53
30m	1h	-0.03	-2.5E-03	0.02	1.00
30m	24h	-0.03	-1.1E-03	0.03	1.00
1h	24h	-0.03	1.3E-03	0.03	1.00
Fumarate					
<5m	15m	-0.01	-1.1E-03	3.3E-03	0.95
<5m	30m	-0.01	-1.2E-03	3.2E-03	0.94
<5m	1h	-0.01	-8.9E-04	3.5E-03	0.98
<5m	24h	-0.01	-3.1E-03	1.3E-03	0.28
15m	30m	-3.6E-03	-6.0E-05	3.5E-03	1.00
15m	1h	-3.3E-03	2.6E-04	3.8E-03	1.00
15m	24h	-0.01	-2.0E-03	1.5E-03	0.52
30m	1h	-3.2E-03	3.2E-04	3.8E-03	1.00
30m	24h	-0.01	-1.9E-03	1.6E-03	0.54
1h	24h	-0.01	-2.3E-03	1.2E-03	0.39
Tyrosine					
<5m	15m	-0.04	-0.01	0.02	0.98
<5m	30m	-0.04	-0.01	0.01	0.63

<5m	1h	-0.06	-0.03	-3.0E-04	0.05
<5m	24h	-0.08	-0.05	-0.02	2.2E-05
15m	30m	-0.03	-0.01	0.01	0.85
15m	1h	-0.05	-0.02	8.6E-05	0.05
15m	24h	-0.07	-0.05	-0.02	2.5E-06
30m	1h	-0.04	-0.01	0.01	0.40
30m	24h	-0.06	-0.04	-0.01	1.5E-04
1h	24h	-0.05	-0.02	1.2E-04	0.05
Phenylalanine					
<5m	15m	-0.14	-0.03	0.08	0.93
<5m	30m	-0.16	-0.06	0.05	0.58
<5m	1h	-0.22	-0.11	-2.3E-03	0.04
<5m	24h	-0.30	-0.19	-0.09	2.0E-05
15m	30m	-0.11	-0.03	0.06	0.91
15m	1h	-0.16	-0.08	0.01	0.09
15m	24h	-0.25	-0.16	-0.08	7.1E-06
30m	1h	-0.14	-0.05	0.03	0.44
30m	24h	-0.22	-0.14	-0.05	2.0E-04
1h	24h	-0.17	-0.08	8.5E-04	0.05
Histidine					
<5m	15m	-0.02	-0.01	0.01	0.92
<5m	30m	-0.02	-3.3E-03	0.01	0.98
<5m	1h	-0.02	-0.01	0.01	0.65
<5m	24h	-0.01	0.01	0.02	0.78
15m	30m	-0.01	1.7E-03	0.02	1.00
15m	1h	-0.02	-3.2E-03	0.01	0.96
15m	24h	0.00	0.01	0.03	0.10
30m	1h	-0.02	-4.9E-03	0.01	0.84
30m	24h	-3.0E-03	0.01	0.02	0.21
1h	24h	1.9E-03	0.02	0.03	0.02
Hypoxanthine					
<5m	15m	-0.03	0.02	0.07	0.82
<5m	30m	-0.04	0.01	0.06	0.99
<5m	1h	-0.04	0.01	0.06	0.99
<5m	24h	-0.08	-0.03	0.02	0.46
15m	30m	-0.05	-0.01	0.03	0.96

15m	1h	-0.05	-0.01	0.03	0.93
15m	24h	-0.09	-0.05	-0.01	0.01
30m	1h	-0.04	-1.5E-03	0.04	1.00
30m	24h	-0.08	-0.04	4.3E-04	0.05
1h	24h	-0.08	-0.04	1.9E-03	0.07
Inosine					
<5m	15m	-0.02	-0.01	0.01	0.92
<5m	30m	-0.01	0.01	0.03	0.89
<5m	1h	-0.01	0.01	0.03	0.78
<5m	24h	-0.01	4.3E-03	0.02	0.97
15m	30m	-3.2E-03	0.01	0.03	0.19
15m	1h	-1.7E-03	0.01	0.03	0.11
15m	24h	-0.01	0.01	0.03	0.36
30m	1h	-0.01	1.5E-03	0.02	1.00
30m	24h	-0.02	-2.0E-03	0.01	1.00
1h	24h	-0.02	-3.5E-03	0.01	0.97
Formate					
<5m	15m	-7.9E-06	0.01	0.02	0.05
<5m	30m	2.9E-03	0.01	0.02	2.7E-03
<5m	1h	0.01	0.02	0.02	1.2E-05
<5m	24h	0.01	0.02	0.03	1.9E-08
15m	30m	-3.9E-03	2.9E-03	0.01	0.76
15m	1h	3.6E-04	0.01	0.01	0.03
15m	24h	0.01	0.01	0.02	3.7E-05
30m	1h	-2.5E-03	4.2E-03	0.01	0.41
30m	24h	2.3E-03	0.01	0.02	2.9E-03
1h	24h	-1.9E-03	4.7E-03	0.01	0.29
Niacinamide					
<5m	15m	2.5E-04	0.01	0.01	0.04
<5m	30m	1.4E-03	0.01	0.02	0.01
<5m	1h	6.3E-04	0.01	0.01	0.03
<5m	24h	-9.8E-04	0.01	0.01	0.13
15m	30m	-4.4E-03	1.1E-03	0.01	0.98
15m	1h	-0.01	3.4E-04	0.01	1.00
15m	24h	-0.01	-1.3E-03	4.2E-03	0.97
30m	1h	-0.01	-8.1E-04	4.6E-03	0.99

30m	24h	-0.01	-2.4E-03	3.0E-03	0.73
1h	24h	-0.01	-1.6E-03	3.8E-03	0.92

[a]VLDL: Very low-density lipoprotein
[b]3-HB: 3-hydroxybutyrate
[c]PC: Phosphocholine
[d]GPC: Glycerophosphocholine

Table S3. Tukey-Kramer Post-Hoc Results for Fetal Surface of Placenta

VLDL					
Group 1	Group 2	Lower CI	Estimate	Upper CI	p-value
<5m	15m	-0.45	-0.07	0.32	0.99
<5m	30m	-0.39	-7.7E-04	0.39	1.00
<5m	1h	-0.35	0.04	0.42	1.00
<5m	24h	-0.14	0.25	0.63	0.39
15m	30m	-0.25	0.06	0.38	0.98
15m	1h	-0.21	0.10	0.42	0.90
15m	24h	2.3E-04	0.31	0.63	0.05
30m	1h	-0.27	0.04	0.35	1.00
30m	24h	-0.06	0.25	0.56	0.18
1h	24h	-0.10	0.21	0.52	0.33
Isoleucine					
<5m	15m	-0.05	-0.01	0.02	0.73
<5m	30m	-0.04	-0.01	0.02	0.91
<5m	1h	-0.05	-0.01	0.02	0.80
<5m	24h	-0.06	-0.03	5.0E-03	0.13
15m	30m	-0.02	4.8E-03	0.03	0.99
15m	1h	-0.03	1.6E-03	0.03	1.00
15m	24h	-0.04	-0.01	0.01	0.65
30m	1h	-0.03	-3.1E-03	0.02	1.00
30m	24h	-0.04	-0.02	0.01	0.34
1h	24h	-0.04	-0.01	0.01	0.54
Leucine					
<5m	15m	-0.25	-0.06	0.13	0.92
<5m	30m	-0.20	-0.01	0.18	1.00
<5m	1h	-0.20	-0.01	0.18	1.00
<5m	24h	-0.25	-0.05	0.14	0.93
15m	30m	-0.11	0.05	0.20	0.93
15m	1h	-0.10	0.05	0.21	0.89

15m	24h	-0.15	3.8E-03	0.16	1.00
30m	1h	-0.15	0.01	0.16	1.00
30m	24h	-0.20	-0.04	0.11	0.94
1h	24h	-0.20	-0.05	0.11	0.91
Valine					
<5m	15m	-0.08	2.8E-03	0.08	1.00
<5m	30m	-0.08	2.5E-03	0.08	1.00
<5m	1h	-0.07	0.01	0.09	1.00
<5m	24h	-0.11	-0.02	0.06	0.91
15m	30m	-0.07	-3.9E-04	0.07	1.00
15m	1h	-0.06	4.7E-03	0.07	1.00
15m	24h	-0.09	-0.03	0.04	0.76
30m	1h	-0.06	0.01	0.07	1.00
30m	24h	-0.09	-0.03	0.04	0.77
1h	24h	-0.10	-0.03	0.03	0.63
3-HB					
<5m	15m	-0.20	0.03	0.25	1.00
<5m	30m	-0.24	-0.02	0.21	1.00
<5m	1h	-0.24	-0.02	0.20	1.00
<5m	24h	-0.21	0.02	0.24	1.00
15m	30m	-0.22	-0.04	0.14	0.97
15m	1h	-0.23	-0.05	0.13	0.95
15m	24h	-0.19	-0.01	0.17	1.00
30m	1h	-0.18	-0.01	0.17	1.00
30m	24h	-0.15	0.03	0.21	0.99
1h	24h	-0.14	0.04	0.22	0.98
Lactate					
<5m	15m	-5.98	-2.21	1.56	0.48
<5m	30m	-8.05	-4.30	-0.56	0.02
<5m	1h	-10.31	-6.57	-2.82	3.9E-05
<5m	24h	-17.99	-14.24	-10.50	9.9E-09
15m	30m	-5.14	-2.09	0.95	0.32
15m	1h	-7.41	-4.36	-1.31	1.2E-03
15m	24h	-15.08	-12.04	-8.99	9.9E-09
30m	1h	-5.28	-2.27	0.75	0.23
30m	24h	-12.96	-9.94	-6.93	9.9E-09

1h	24h	-10.69	-7.68	-4.66	1.2E-08
Alanine					
<5m	15m	-0.18	0.03	0.23	1.00
<5m	30m	-0.18	0.02	0.23	1.00
<5m	1h	-0.20	0.01	0.21	1.00
<5m	24h	-0.31	-0.10	0.10	0.62
15m	30m	-0.17	-3.7E-03	0.16	1.00
15m	1h	-0.18	-0.02	0.15	1.00
15m	24h	-0.30	-0.13	0.04	0.20
30m	1h	-0.18	-0.01	0.15	1.00
30m	24h	-0.29	-0.13	0.04	0.22
1h	24h	-0.28	-0.11	0.05	0.33
Acetate					
<5m	15m	-0.06	-0.01	0.05	1.00
<5m	30m	-0.07	-0.02	0.04	0.92
<5m	1h	-0.07	-0.01	0.04	0.96
<5m	24h	-0.07	-0.01	0.04	0.96
15m	30m	-0.05	-0.01	0.04	0.98
15m	1h	-0.05	-0.01	0.04	0.99
15m	24h	-0.05	-0.01	0.04	0.99
30m	1h	-0.04	2.8E-03	0.05	1.00
30m	24h	-0.04	2.8E-03	0.05	1.00
1h	24h	-0.04	-7.0E-05	0.04	1.00
Acetone					
<5m	15m	-0.03	0.01	0.05	0.99
<5m	30m	-0.04	-8.1E-04	0.04	1.00
<5m	1h	-0.04	0.01	0.05	0.99
<5m	24h	-0.01	0.03	0.07	0.18
15m	30m	-0.04	-0.01	0.03	0.96
15m	1h	-0.03	-9.9E-04	0.03	1.00
15m	24h	-0.01	0.03	0.06	0.21
30m	1h	-0.03	0.01	0.04	0.98
30m	24h	6.7E-04	0.03	0.07	0.04
1h	24h	-0.01	0.03	0.06	0.17
Glutamate					

<5m	15m	-0.23	0.11	0.45	0.90
<5m	30m	-0.31	0.03	0.36	1.00
<5m	1h	-0.31	0.03	0.37	1.00
<5m	24h	-0.08	0.26	0.60	0.23
15m	30m	-0.36	-0.09	0.19	0.91
15m	1h	-0.36	-0.08	0.19	0.92
15m	24h	-0.13	0.15	0.42	0.59
30m	1h	-0.27	4.7E-03	0.28	1.00
30m	24h	-0.04	0.23	0.50	0.14
1h	24h	-0.05	0.23	0.50	0.15
[redacted]				[redacted]	
Succinate					
<5m	15m	-0.06	8.4E-04	0.06	1.00
<5m	30m	-0.07	-0.01	0.04	0.95
<5m	1h	-0.10	-0.04	0.01	0.22
<5m	24h	-0.14	-0.08	-0.03	6.1E-04
15m	30m	-0.06	-0.02	0.03	0.88
15m	1h	-0.09	-0.04	1.7E-03	0.07
15m	24h	-0.13	-0.08	-0.04	1.1E-05
30m	1h	-0.07	-0.03	0.02	0.41
30m	24h	-0.11	-0.07	-0.02	4.1E-04
1h	24h	-0.09	-0.04	4.1E-03	0.09
[redacted]				[redacted]	
Glutamine					
<5m	15m	-0.08	0.03	0.14	0.94
<5m	30m	-0.12	-4.5E-03	0.11	1.00
<5m	1h	-0.14	-0.02	0.09	0.98
<5m	24h	-0.09	0.02	0.13	0.99
15m	30m	-0.13	-0.04	0.06	0.82
15m	1h	-0.15	-0.05	0.04	0.46
15m	24h	-0.10	-0.01	0.08	1.00
30m	1h	-0.11	-0.02	0.07	0.98
30m	24h	-0.06	0.02	0.11	0.94
1h	24h	-0.05	0.04	0.13	0.65
[redacted]				[redacted]	
Citrate					
<5m	15m	-0.06	0.01	0.08	1.00
<5m	30m	-0.09	-0.02	0.05	0.96
<5m	1h	-0.10	-0.03	0.04	0.84

<5m	24h	-3.4E-03	0.07	0.14	0.07
15m	30m	-0.08	-0.02	0.03	0.76
15m	1h	-0.09	-0.03	0.02	0.49
15m	24h	2.6E-03	0.06	0.12	0.04
30m	1h	-0.07	-0.01	0.05	0.99
30m	24h	0.03	0.08	0.14	6.9E-04
1h	24h	0.04	0.09	0.15	1.3E-04
Aspartate					
<5m	15m	-0.04	-0.01	0.02	0.98
<5m	30m	-0.04	-0.01	0.02	0.98
<5m	1h	-0.03	-0.01	0.02	0.99
<5m	24h	-0.03	-1.2E-03	0.03	1.00
15m	30m	-0.02	-1.4E-05	0.02	1.00
15m	1h	-0.02	1.0E-03	0.02	1.00
15m	24h	-0.02	4.8E-03	0.03	0.98
30m	1h	-0.02	1.0E-03	0.02	1.00
30m	24h	-0.02	4.8E-03	0.03	0.98
1h	24h	-0.02	3.8E-03	0.03	0.99
Asparagine					
<5m	15m	-0.04	-0.01	0.03	0.98
<5m	30m	-0.03	1.3E-03	0.03	1.00
<5m	1h	-0.03	3.9E-03	0.04	1.00
<5m	24h	-0.01	0.02	0.05	0.30
15m	30m	-0.02	0.01	0.03	0.93
15m	1h	-0.02	0.01	0.04	0.83
15m	24h	2.4E-03	0.03	0.05	0.02
30m	1h	-0.02	2.6E-03	0.03	1.00
30m	24h	-4.8E-03	0.02	0.05	0.16
1h	24h	-0.01	0.02	0.04	0.28
Lysine					
<5m	15m	-0.07	-0.01	0.05	1.00
<5m	30m	-0.04	0.02	0.08	0.92
<5m	1h	-0.04	0.02	0.08	0.89
<5m	24h	-0.05	0.01	0.07	0.99
15m	30m	-0.02	0.03	0.08	0.57
15m	1h	-0.02	0.03	0.08	0.51

15m	24h	-0.03	0.02	0.07	0.86
30m	1h	-0.05	1.8E-03	0.05	1.00
30m	24h	-0.06	-0.01	0.04	0.99
1h	24h	-0.06	-0.01	0.04	0.97
Creatine					
<5m	15m	-0.11	0.32	0.75	0.24
<5m	30m	-0.05	0.38	0.80	0.11
<5m	1h	-0.13	0.30	0.72	0.30
<5m	24h	-0.20	0.22	0.65	0.60
15m	30m	-0.29	0.06	0.40	0.99
15m	1h	-0.37	-0.02	0.32	1.00
15m	24h	-0.44	-0.10	0.25	0.94
30m	1h	-0.42	-0.08	0.26	0.97
30m	24h	-0.50	-0.16	0.19	0.71
1h	24h	-0.42	-0.08	0.27	0.97
Choline					
<5m	15m	-1.37	-0.41	0.55	0.76
<5m	30m	-1.82	-0.86	0.09	0.09
<5m	1h	-1.91	-0.95	-3.3E-04	0.05
<5m	24h	-3.38	-2.43	-1.48	1.2E-08
15m	30m	-1.23	-0.45	0.32	0.49
15m	1h	-1.32	-0.54	0.23	0.30
15m	24h	-2.79	-2.02	-1.24	1.1E-08
30m	1h	-0.86	-0.09	0.68	1.00
30m	24h	-2.33	-1.56	-0.80	1.3E-06
1h	24h	-2.24	-1.47	-0.71	5.5E-06
PC					
<5m	15m	0.01	0.24	0.48	0.04
<5m	30m	-0.03	0.20	0.44	0.12
<5m	1h	-0.04	0.20	0.43	0.14
<5m	24h	0.27	0.50	0.73	3.5E-07
15m	30m	-0.23	-0.04	0.15	0.98
15m	1h	-0.23	-0.04	0.15	0.97
15m	24h	0.07	0.26	0.45	2.4E-03
30m	1h	-0.19	-4.3E-03	0.18	1.00
30m	24h	0.11	0.30	0.49	0.00

1h	24h	0.11	0.30	0.49	0.00
GPC					
<5m	15m	-0.27	0.48	1.24	0.39
<5m	30m	-0.25	0.49	1.24	0.36
<5m	1h	0.06	0.80	1.55	0.03
<5m	24h	0.72	1.46	2.21	3.5E-06
15m	30m	-0.60	0.01	0.62	1.00
15m	1h	-0.29	0.32	0.93	0.59
15m	24h	0.37	0.98	1.59	1.9E-04
30m	1h	-0.29	0.31	0.91	0.61
30m	24h	0.37	0.97	1.57	1.9E-04
1h	24h	0.06	0.66	1.26	0.02
Taurine					
<5m	15m	-0.21	0.25	0.71	0.56
<5m	30m	-0.30	0.16	0.62	0.87
<5m	1h	-0.09	0.37	0.83	0.18
<5m	24h	-0.09	0.37	0.83	0.18
15m	30m	-0.47	-0.09	0.28	0.96
15m	1h	-0.26	0.12	0.49	0.91
15m	24h	-0.26	0.12	0.49	0.91
30m	1h	-0.16	0.21	0.58	0.53
30m	24h	-0.16	0.21	0.58	0.52
1h	24h	-0.37	1.5E-03	0.37	1.00
Glucose					
<5m	15m	-0.26	0.19	0.64	0.76
<5m	30m	-0.33	0.12	0.57	0.95
<5m	1h	-0.08	0.37	0.82	0.16
<5m	24h	-0.06	0.39	0.84	0.12
15m	30m	-0.44	-0.07	0.29	0.98
15m	1h	-0.19	0.18	0.54	0.66
15m	24h	-0.17	0.20	0.56	0.57
30m	1h	-0.11	0.25	0.61	0.30
30m	24h	-0.09	0.27	0.63	0.23
1h	24h	-0.34	0.02	0.38	1.00
Myo-inositol					

<5m	15m	-0.51	-0.02	0.48	1.00
<5m	30m	-0.60	-0.11	0.37	0.97
<5m	1h	-0.59	-0.10	0.38	0.98
<5m	24h	-0.55	-0.07	0.42	1.00
15m	30m	-0.49	-0.10	0.30	0.96
15m	1h	-0.48	-0.09	0.31	0.97
15m	24h	-0.45	-0.05	0.35	1.00
30m	1h	-0.38	0.01	0.40	1.00
30m	24h	-0.35	0.05	0.44	1.00
1h	24h	-0.36	0.04	0.43	1.00
Glycine					
<5m	15m	-0.14	0.01	0.15	1.00
<5m	30m	-0.15	-0.01	0.13	1.00
<5m	1h	-0.14	-2.3E-03	0.14	1.00
<5m	24h	-0.27	-0.13	0.01	0.09
15m	30m	-0.13	-0.02	0.10	1.00
15m	1h	-0.12	-0.01	0.11	1.00
15m	24h	-0.25	-0.14	-0.02	0.01
30m	1h	-0.11	0.01	0.12	1.00
30m	24h	-0.23	-0.12	-0.01	0.03
1h	24h	-0.24	-0.13	-0.01	0.02
Glycerol					
<5m	15m	-0.09	-0.02	0.04	0.87
<5m	30m	-0.11	-0.04	0.02	0.30
<5m	1h	-0.12	-0.05	0.01	0.15
<5m	24h	-0.31	-0.24	-0.18	9.9E-09
15m	30m	-0.07	-0.02	0.03	0.75
15m	1h	-0.08	-0.03	0.02	0.46
15m	24h	-0.27	-0.22	-0.17	9.9E-09
30m	1h	-0.06	-0.01	0.04	0.99
30m	24h	-0.25	-0.20	-0.15	9.9E-09
1h	24h	-0.24	-0.19	-0.14	9.9E-09
Serine					
<5m	15m	-0.27	0.07	0.41	0.97
<5m	30m	-0.34	-0.01	0.33	1.00
<5m	1h	-0.21	0.12	0.46	0.84

<5m	24h	-0.31	0.03	0.36	1.00
15m	30m	-0.36	-0.08	0.19	0.92
15m	1h	-0.22	0.05	0.33	0.99
15m	24h	-0.32	-0.05	0.23	0.99
30m	1h	-0.14	0.13	0.40	0.66
30m	24h	-0.24	0.03	0.30	1.00
1h	24h	-0.37	-0.10	0.17	0.85
Threonine					
<5m	15m	-0.03	0.00	0.03	1.00
<5m	30m	-0.04	-0.01	0.02	0.96
<5m	1h	-0.03	-1.3E-03	0.03	1.00
<5m	24h	4.8E-03	0.04	0.07	0.01
15m	30m	-0.03	-0.01	0.02	0.98
15m	1h	-0.02	1.3E-03	0.03	1.00
15m	24h	0.01	0.04	0.06	4.6E-04
30m	1h	-0.02	0.01	0.03	0.95
30m	24h	0.02	0.04	0.07	4.0E-05
1h	24h	0.01	0.04	0.06	6.8E-04
Glutathione					
<5m	15m	-0.04	-0.01	0.02	0.84
<5m	30m	-0.04	-0.01	0.02	0.91
<5m	1h	-0.04	-0.02	0.01	0.51
<5m	24h	-0.02	0.01	0.04	0.78
15m	30m	-0.02	1.8E-03	0.02	1.00
15m	1h	-0.03	-0.01	0.02	0.96
15m	24h	-6.6E-04	0.02	0.04	0.06
30m	1h	-0.03	-0.01	0.01	0.89
30m	24h	-2.3E-03	0.02	0.04	0.10
1h	24h	4.9E-03	0.03	0.05	0.01
Uracil					
<5m	15m	-0.02	1.9E-03	0.02	1.00
<5m	30m	-0.02	3.6E-03	0.02	0.99
<5m	1h	-0.02	2.9E-03	0.02	1.00
<5m	24h	-0.04	-0.02	1.7E-03	0.09
15m	30m	-0.02	1.7E-03	0.02	1.00
15m	1h	-0.02	1.0E-03	0.02	1.00

15m	24h	-0.04	-0.02	-4.0E-03	0.01
30m	1h	-0.02	-7.0E-04	0.02	1.00
30m	24h	-0.04	-0.02	-0.01	2.6E-03
1h	24h	-0.04	-0.02	-0.01	3.8E-03
Uridine					
<5m	15m	-0.03	-0.01	0.02	0.97
<5m	30m	-0.04	-0.02	0.01	0.55
<5m	1h	-0.04	-0.01	0.02	0.83
<5m	24h	-0.03	-0.01	0.02	0.97
15m	30m	-0.03	-0.01	0.01	0.80
15m	1h	-0.03	-4.2E-03	0.02	0.99
15m	24h	-0.02	1.7E-04	0.02	1.00
30m	1h	-0.02	4.8E-03	0.03	0.98
30m	24h	-0.01	0.01	0.03	0.79
1h	24h	-0.02	4.4E-03	0.03	0.98
Fumarate					
<5m	15m	-0.01	-1.0E-03	3.5E-03	0.97
<5m	30m	-0.01	-8.3E-04	3.6E-03	0.99
<5m	1h	-0.01	-2.0E-03	2.4E-03	0.71
<5m	24h	-0.01	-0.01	-9.2E-04	0.01
15m	30m	-3.4E-03	2.0E-04	3.8E-03	1.00
15m	1h	-4.6E-03	-1.0E-03	2.6E-03	0.94
15m	24h	-0.01	-4.3E-03	-7.2E-04	0.01
30m	1h	-4.8E-03	-1.2E-03	2.4E-03	0.88
30m	24h	-0.01	-4.5E-03	-9.6E-04	0.01
1h	24h	-0.01	-3.3E-03	2.4E-04	0.08
Tyrosine					
<5m	15m	-0.02	1.7E-03	0.03	1.00
<5m	30m	-0.02	0.01	0.03	0.91
<5m	1h	-0.02	0.01	0.03	0.91
<5m	24h	-0.03	-4.3E-03	0.02	0.99
15m	30m	-0.01	0.01	0.02	0.93
15m	1h	-0.01	0.01	0.02	0.93
15m	24h	-0.03	-0.01	0.01	0.91
30m	1h	-0.02	1.2E-05	0.02	1.00
30m	24h	-0.03	-0.01	0.01	0.45

1h	24h	-0.03	-0.01	0.01	0.45
Phenylalanine					
<5m	15m	-0.10	-0.01	0.07	1.00
<5m	30m	-0.06	0.02	0.11	0.94
<5m	1h	-0.07	0.02	0.10	0.99
<5m	24h	-0.11	-0.03	0.06	0.90
15m	30m	-0.03	0.04	0.11	0.61
15m	1h	-0.04	0.03	0.10	0.80
15m	24h	-0.09	-0.02	0.05	0.97
30m	1h	-0.08	-0.01	0.06	1.00
30m	24h	-0.12	-0.05	0.02	0.23
1h	24h	-0.11	-0.04	0.03	0.41
Histidine					
<5m	15m	-0.01	-4.6E-05	0.01	1.00
<5m	30m	-0.01	2.0E-03	0.01	0.99
<5m	1h	-0.01	2.6E-03	0.01	0.98
<5m	24h	-0.01	4.3E-03	0.02	0.86
15m	30m	-0.01	2.0E-03	0.01	0.98
15m	1h	-0.01	2.7E-03	0.01	0.95
15m	24h	-0.01	4.4E-03	0.01	0.74
30m	1h	-0.01	6.8E-04	0.01	1.00
30m	24h	-0.01	2.4E-03	0.01	0.96
1h	24h	-0.01	1.7E-03	0.01	0.99
Hypoxanthine					
<5m	15m	-0.03	0.02	0.08	0.74
<5m	30m	-0.01	0.05	0.10	0.12
<5m	1h	-0.03	0.02	0.08	0.76
<5m	24h	-0.03	0.02	0.07	0.81
15m	30m	-0.02	0.02	0.07	0.59
15m	1h	-0.04	-7.8E-04	0.04	1.00
15m	24h	-0.05	-2.6E-03	0.04	1.00
30m	1h	-0.07	-0.02	0.02	0.55
30m	24h	-0.07	-0.03	0.02	0.47
1h	24h	-0.04	-1.8E-03	0.04	1.00
Inosine					

<5m	15m	-0.03	-2.8E-03	0.02	1.00
<5m	30m	-0.04	-0.01	0.01	0.72
<5m	1h	-0.03	-0.01	0.02	0.92
<5m	24h	-0.03	-0.01	0.02	0.85
15m	30m	-0.03	-0.01	0.01	0.78
15m	1h	-0.02	-4.5E-03	0.02	0.97
15m	24h	-0.03	-0.01	0.01	0.91
30m	1h	-0.02	3.7E-03	0.02	0.99
30m	24h	-0.02	2.1E-03	0.02	1.00
1h	24h	-0.02	-1.6E-03	0.02	1.00
[redacted]					
Formate					
<5m	15m	-0.02	5.0E-03	0.03	0.96
<5m	30m	-0.01	0.01	0.03	0.92
<5m	1h	-0.02	-1.4E-03	0.02	1.00
<5m	24h	-0.01	0.01	0.03	0.63
15m	30m	-0.02	1.0E-03	0.02	1.00
15m	1h	-0.02	-0.01	0.01	0.82
15m	24h	-0.01	0.01	0.02	0.91
30m	1h	-0.02	-0.01	0.01	0.71
30m	24h	-0.01	4.0E-03	0.02	0.96
1h	24h	-4.7E-03	0.01	0.03	0.29
[redacted]					
Niacinamide					
<5m	15m	-9.9E-04	0.01	0.01	0.14
<5m	30m	1.1E-03	0.01	0.01	0.01
<5m	1h	3.8E-04	0.01	0.01	0.03
<5m	24h	-2.8E-03	3.6E-03	0.01	0.52
15m	30m	-3.2E-03	2.0E-03	0.01	0.82
15m	1h	-3.9E-03	1.3E-03	0.01	0.96
15m	24h	-0.01	-1.8E-03	3.4E-03	0.87
30m	1h	-0.01	-6.8E-04	4.5E-03	1.00
30m	24h	-0.01	-3.8E-03	1.3E-03	0.24
1h	24h	-0.01	-3.2E-03	2.0E-03	0.44

[a]VLDL: Very low-density lipoprotein
[b]3-HB: 3-hydroxybutyrate
[c]PC: Phosphocholine
[d]GPC: Glycerophosphocholine
